# Bayesian Analysis of the Ordinal Markov Random Field

**DOI:** 10.1017/psy.2024.4

**Published:** 2025-01-03

**Authors:** Maarten Marsman, Don van den Bergh, Jonas M. B. Haslbeck

**Affiliations:** Department of Psychology, University of Amsterdam, Amsterdam, Netherlands; Department of Clinical Psychological Science, Maastricht University, Maastricht, Netherlands

**Keywords:** Bayesian variable selection, graphical model, Markov random field, network psychometrics, ordinal variables

## Abstract

Multivariate analysis using graphical models is rapidly gaining ground in psychology. In particular, Markov random field (MRF) graphical models have become popular because their graph structure reflects the conditional associations between psychological variables. Despite the fact that most psychological variables are assessed on an ordinal scale, the analysis of MRFs for ordinal variables has received little attention in the psychometric literature. To fill this gap, we present an MRF for ordinal data that so far has not been considered in network psychometrics. We present statistical methodology to test the structure of the proposed MRF, which requires us to determine the plausibility of the opposing hypotheses of conditional dependence and independence. To this end, we develop a Bayesian approach using the inclusion Bayes factor to quantify the (lack of) evidence for a given edge. We use a Bayesian variable selection approach to model the inclusion and exclusion of edges in the network, and Bayesian model averaging to compare network structures with and without the given edge. We provide an implementation in the new R package bgms, evaluate its performance in simulations, and illustrate it with empirical data.

## 1 Introduction

Multivariate analysis using graphical models has received much attention in the recent psychological and psychometric literature (e.g., Contreras et al., 2019; Marsman & Rhemtulla, 2022; Robinaugh et al., 2020). Most of these graphical models are Markov random field (MRF) models, whose graph structure reflects the conditional associations between variables (Kindermann & Snell, 1980). In these models, a missing edge between two variables in the network implies that these variables are independent, given the remaining variables (Lauritzen, 2004). In other words, the remaining variables of the network fully account for the potential association between the unconnected variables. The methodology for analyzing MRFs for binary, unordered categorical, and continuous variables and their combinations has been well developed (e.g., Epskamp et al., 2018b; Haslbeck & Waldorp, 2020; Marsman et al., 2022; Mohammadi & Wit, 2015; van Borkulo et al., 2014; Williams, 2021). The analysis of MRFs for ordinal variables, on the other hand, has received little attention in the psychometric literature, despite the abundance of ordinal variables in psychological data (e.g., Isvoranu & Epskamp, 2023; Johal & Rhemtulla, 2023). Since there is no MRF for ordinal variables, researchers must use misspecified models to analyze their data. Gaussian graphical models (GGMs), which assume continuous data, are a popular solution, although many have cautioned about the problems of using models such as the GGM to analyze ordinal data (e.g., Johal & Rhemtulla, 2023; Liddell & Kruschke, 2018) and that results between GGMs and discrete graphical models may be misaligned (Loh & Wainwright, 2013). Another common solution is to dichotomize ordinal data and analyze them with a network model for binary variables. However, dichotomization thresholds are often chosen ad hoc, and this choice can have a significant impact on the resulting network estimates (e.g., Hoffman et al., 2018). Here, we address this problem by presenting an MRF for ordinal variables and providing a Bayesian procedure to assess its Markov structure.

We present an MRF for ordinal data that has been proposed in both the psychometric and machine learning literature but has so far not been considered in network psychometrics. In psychometrics, Anderson and Vermunt (2000) derived a joint graphical model to manifest ordinal and unobserved continuous variables. By imposing a conditional Gaussian distribution (Lauritzen & Wermuth, 1989) on the latent variables, given the ordinal variables, they were able to derive a log-multiplicative association model for the distribution of the ordinal variables. We will show below that this log-multiplicative association model is an MRF for ordinal variables. Independently of the work of Anderson and Vermunt, in the machine learning literature, Suggala et al. (2017) derived an MRF for ordinal variables via a Hammersley–Clifford style analysis (Besag, 1974), introducing a model for the full conditional distribution for each variable in the network, given the remaining variables, and showing that this leads to a unique joint distribution. We will introduce this ordinal MRF and connect the work of Anderson and Vermunt (2000) and Suggala et al. (2017).

Testing the structure of the MRF requires us to determine the plausibility of the opposing hypotheses of conditional dependence and conditional independence. For example, how plausible are network structures that include the edge between variables 3 and 9 compared to network structures that exclude this edge? Frequentist approaches are limited in this respect, because they can only reject the conditional independence hypothesis but not support it (e.g., Wagenmakers, 2007; Wagenmakers, 2018b; Marsman, et al., 2018). This creates the problem that, if an edge is excluded, we do not know whether this is because the edge is absent in the population, or because we lack the power to reject the null hypothesis of independence. To avoid this problem, we will use a Bayesian approach using Bayes factors (e.g., Kass & Raftery, 1995). Specifically, we use the inclusion Bayes factor (e.g., Huth et al., 2021, 2023), which allows us to quantify how much the data support both conditional dependence—*evidence of edge presence*—and conditional independence —*evidence of edge absence*. It also allows us to conclude that there is limited support for either hypothesis (e.g., Dienes, 2014)—*absence of evidence*.

The inclusion Bayes factor uses Bayesian model averaging (Hinne et al., 2020; Hoeting et al., 1999; Kaplan, 2021) to evaluate how well network structures with or without a given edge predict the data at hand. However, the large number of possible structures to evaluate for these predictions poses a serious challenge to Bayesian model averaging. To address this challenge, we use Bayesian variable selection (Tadesse & Vanucci, 2022) to model the inclusion and exclusion of edges in the network and set up a Markov chain with the posterior distribution of the network structures for the MRF as an invariant distribution (e.g., George & McCulloch, 1993). Our variable selection approach specifies a two-component mixture as the prior for the edge weights of the MRF—a discrete spike and a slab prior (Mitchell & Beauchamp, 1988; Vanucci, 2022)—where one component is a relatively diffuse prior (i.e., the slab), while the other component is a prior that assigns its entire probability mass to zero (i.e., the spike). We use a latent indicator variable, 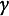 , to assign the edge weight to one of these two components: 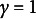 implies that the edge is in the network and assigns a diffuse prior to its corresponding weight. On the other hand, 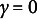 implies that the edge is not in the network and sets the corresponding edge weight to zero. While this approach allows us to explore the large space of possible network structures—configurations of edge indicators—it also introduces two computational challenges. The first challenge is the computational intractability of the MRF due to its complex normalization constant. We adopt a pseudolikelihood approach (Besag, 1975) to address this challenge but also explore the double Metropolis–Hastings (DMH) algorithm (Liang, 2010) as an alternative. The second challenge is that setting edge weights to zero when the edge is absent leads to different parameter dimensions for different network structures, and it is difficult to formulate a proper Markov chain over this probability space. To address this challenge, we use the transdimensional Markov chain method of Gottardo and Raftery (2008). We incorporate these solutions into a Gibbs sampling approach (Geman & Geman, 1984) to sample from the multivariate posterior distribution of the model parameters and edge indicators. The proposed methods are implemented in the software bgms (Marsman et al., 2023), an open-source R package (R Core Team, 2019) that is freely available on CRAN.

This article is organized as follows. In the next section, we will introduce the MRF for ordinal variables and connect the work of Anderson and Vermunt (2000) and Suggala et al. (2017). We will also establish some basic properties of the proposed model. Next, we specify and discuss the components of our Bayesian model, i.e., the prior distributions, in Section 3. We then introduce our Markov chain Monte Carlo edge selection approach in Section 4, where we discuss our pseudolikelihood solution to the intractability of the likelihood, the transdimensional method of Gottardo and Raftery (2008), and the combined Gibbs sampler. In Section 5, we use simulations to demonstrate the consistency of our Bayesian edge selection approach when analyzing binary or ordinal variables and compare it to two existing approaches when analyzing binary variables. In Section 6, we illustrate the added value of the proposed method for applied researchers by reanalyzing a dataset on posttraumatic stress disorder (PTSD). We conclude with relating our model to other multivariate ordinal models and discussing the performance and possible improvements of the proposed Bayesian methodology.

## 2 The Markov random field for ordinal variables

This section introduces and examines the proposed MRF for ordinal variables. We consider two different approaches to establishing the MRF that have not been previously linked. The approach presented by Anderson and Vermunt (2000) shows us that the MRF is related to a classical psychometric model for ordinal variables and how their parameters are related. The approach presented by Suggala et al. (2017) shows us that the MRF is a unique multivariate extension of a particular logit model for ordinal variables. Their work also shows us that we are not aware of other logit models for ordinal variables that have a multivariate extension. Therefore, we will refer to the MRF as the ordinal MRF and accept the risk of finding a new type of ordinal logit that has a multivariate extension.^1^ We will first discuss the two approaches to building the MRF and then examine its Markov properties and parameter identification.

### 2.1 A psychometric approach to the ordinal Markov random field

Anderson and Vermunt (2000) established the MRF in the psychometric literature as the distribution of manifest ordinal variables in contexts containing continuous latent variables. Such relationships between manifest and latent variables are central to psychometrics (e.g., Holland & Rosenbaum, 1986), and the psychometric literature reports various approaches to establishing these relationships. One well-known method is the Dutch identity of Holland (1990), which uses the same underlying assumptions as the approach of Anderson and Vermunt (2000; see Anderson & Yu, 2007, for a discussion). More recently, Marsman et al. (2019) proposed a related method that emerged from the connection between a binary variable MRF and item response theory (IRT) models in network psychometrics (e.g., Epskamp et al., 2018a; Marsman et al., 2018; Marsman et al., 2015). See Hessen (2020) for yet another method. These approaches make the same assumptions about the distribution of unobserved latent variables and thus can all be used to obtain the results of Anderson and Vermunt (2000). Here, we use the general setup of Marsman et al. (2019) because it provides a straightforward strategy for deriving the MRF from an existing IRT model.

Let 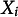 denote an ordinal variable with 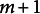 response categories and realizations 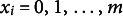 , for 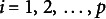 variables.^2^ We assume that the joint distribution for the full vector of *p* response variables 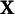 can be modeled using an item response theory (IRT) model, 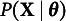 , in which a vector of continuous latent variables 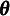 of at most dimension 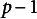 fully accounts for the dependencies between the *p* response variables. In this context, Muraki (1990) proposed the generalized partial credit model (GPCM) for ordinal responses. The multidimensional version of this GPCM is characterized by the following probability of observing category *h* of the ordinal response variable: 

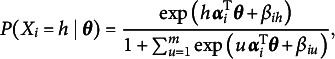

where 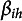 denotes a threshold parameter for category *h* of variable or item *i*, with 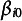 set to zero for identification, and 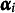 denotes a vector of factor-loadings that relate the latent variables to the response probabilities for item *i*. If we let 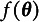 denote the distribution for the latent variables, the full statistical model has the form 

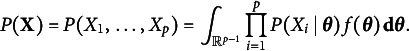

We aim to find an expression for 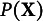 , the marginal distribution of the response variables.

Marsman et al. (2019) showed that if an IRT model is in an exponential family form, we can define a latent variable distribution that allows us to analytically express the marginal distribution 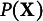 . We define the exponential family form of the IRT model as follows: 

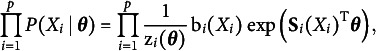

where 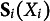 is a (possibly vector-valued) statistic that is sufficient for the (possibly vector-valued) latent variable 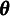 , 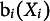 is a base measure that does not depend on the latent variable, and 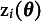 is a normalizing constant that does not depend on the observed response variable and ensures that the probabilities add up to one. The normalizing constant is equal to 

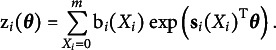

We can express the multidimensional GPCM in this form. That is, we define the sufficient statistic as 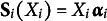 , the logarithm of the base measure as 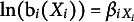 , and the normalizing constant as 

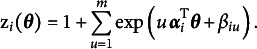

We can now define a latent variable distribution as follows: 

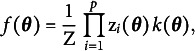

where 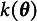 is a density function and 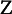 is a normalizing constant. The normalizing constant is equal to 
(1)

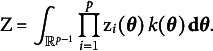



The proposed distribution 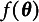 is valid for any density function 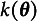 for which 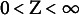 . Corollary 1 in Marsman et al. (2019) shows that if we assume that the density 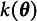 is a multivariate normal distribution with a mean vector 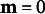 and covariance matrix 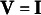 , the identity matrix, we find the following expression for the marginal distribution: 

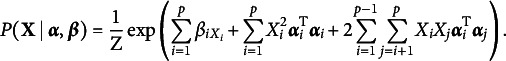

From here, we recognize the quadratic form 
(2)

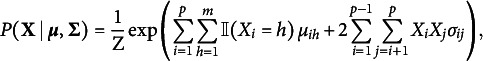

where 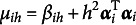 and 
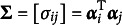
 , and 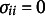 , for 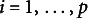 . The marginal distribution 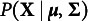 is the desired MRF and equal to Eq. (20) in Anderson and Vermunt (2000; see also Theorem 2 in Hessen, 2020). The normalizing constant that we defined in Eq. (1) can now be restated as 
(3)

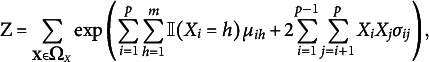

where 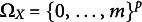 denotes the space of possible response patterns. Observe that when 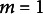 , the MRF in Eq. (2) simplifies to the Ising model (Cox, 1972; Ising, 1925).

### 2.2 A Hammersley–Clifford approach to the ordinal Markov random field

Suggala et al. (2017) established the MRF in the statistical learning literature as a unique multivariate distribution, consistent with a specific full conditional distribution for ordinal variables. They extended the work of Yang et al. (2012), who provided a method for constructing a multivariate graphical model from univariate full conditional distributions. These approaches follow the Hammersley–Clifford approach advocated by Besag (1974). Suggala et al. (2017) set out to use the method proposed by Yang et al. (2012) to establish the multivariate distribution consistent with standard univariate models for ordinal variables based on cumulative, continuous, and consecutive ratio logits (Agresti, 2010, 2018). However, the method of Yang et al. (2012) assumes that the conditional distributions are in the exponential family. Suggala et al. (2017) show that the cumulative and continuation-based logits do not belong to this class of models and consequently do not yield a multivariate distribution. On the other hand, the consecutive-based logit models belong to the exponential family and are also consistent with a multivariate distribution. These results are discussed next.

The Hammersley–Clifford theorem states that if a multivariate distribution 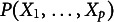 is consistent with certain full conditional distributions 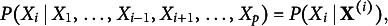 then it is the only multivariate distribution consistent with these conditional distributions. Besag (1974) suggested using this idea to determine whether there is a multivariate distribution that is consistent with certain univariate full conditional distributions. Suggala et al. (2017) use this idea to analyze three popular models for ordinal variables: the cumulative ratio, the continuation ratio, and the consecutive ratio (or adjacent category) logits (Agresti, 2010, 2018). Theorem 3 in Suggala et al. (2017) shows that there is a multivariate distribution consistent with the consecutive ratio or adjacent categories logit, 

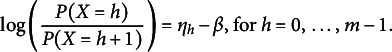



We use this logit to define the full conditional of a node *i* given the remaining variables 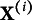 , by setting 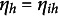 and 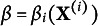 , such that the full conditional is of the form 

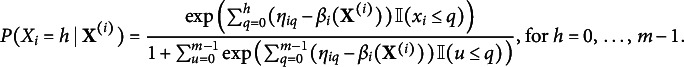

Theorem 3 in Suggala et al. (2017) shows that the multivariate distribution that is consistent with this full conditional is equal to 

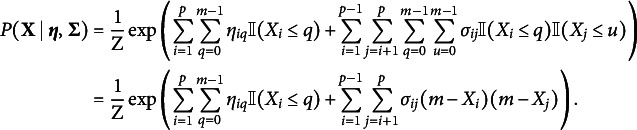

When we redefine 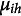 as 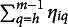 and 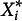 as 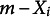 , this multivariate distribution is equal to the MRF in Eq. (2). Thus, the proposed MRF in Eq. (2) is a multivariate extension of the adjacent category model.

Suggala et al. (2017) showed that no multivariate distribution is consistent with either of the other two models for ordinal variables. The cumulative ratio or proportional odds is defined as 

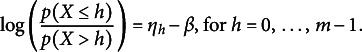

This logit model is similar in nature to the probit model (Guo et al., 2015), in which the realizations of the ordinal variable correspond to adjacent intervals of an underlying latent variable. Theorem 1 in Suggala et al. (2017) shows that no multivariate distribution is consistent with having this logit model as full-conditional distribution. Furthermore, their Theorem 2 shows that there is also no multivariate distribution with full conditionals based on the continuation ratio: 

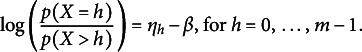

This implies that the proposed MRF is a rather unique multivariate model for ordinal variables.

### 2.3 Markov properties

The model in Eq. (2) is a Markov random field model in which the manifest variables are viewed as nodes of a graph or network whose structure is described by the matrix of pairwise interaction effects or edge weights 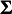 . If 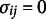 , then the variables *i* and *j* do not directly interact in the network. In Appendix A, we show that the model satisfies a global Markov property. A convenient feature of this Markov property is that the structure of the graph—characterized by the parameters 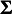 —represents the conditional dependence relations between variables in the network. This means that if 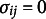 , variables *i* and *j* are conditionally independent given the rest of the variables in the network. In other words, the remaining variables 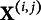 fully account for a possible association between 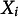 and 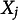 .

One way to examine the properties of the model is to focus on its local properties, such as those implied by its full conditional distribution, the distribution of one or more variables given all other variables in the network. Appendix A shows the general form of conditional distributions for the ordinal MRF. Here we focus on the conditional distribution of one of the variables in the network: 
(4)

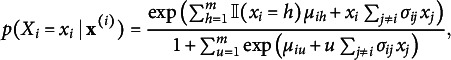

which is similar to a logistic regression for ordinal outcomes using the remaining variables as predictors. Here we see that when the interaction between variables *i* and *j* is positive, responding in a higher category for variable *i* also tends to lead to responding in a higher category for variable *j*. And if there is no direct relationship between variables *i* and *j* (i.e., 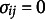 ), we see that variable *j* drops from the conditional distribution of variable *i*, a consequence of the Markov property of the model. In the absence of interactions, i.e., 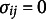 for all 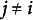 , the full conditional distribution simplifies to a categorical distribution (Agresti, 2018) with parameters 

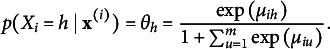

The category threshold parameters for the ordinal MRF thus express the tendency of ordinal response variables that cannot be explained by the remaining variables in the graph or network.

### 2.4 Parameter identification and interpretation

The model is in the exponential family and has sufficient statistics for each parameter. Here we use this to establish the identification of the model parameters. Let 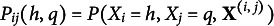 denote the probability that variables *i* and *j* in the network take values *h* and *q*, respectively. Then we can see that the interaction parameter 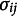 is related to the ratio of the adjacent category odds of the two ordinates: 

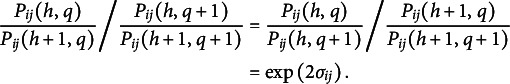

Thus, there is a direct mapping between the manifest variable—the ratio of observed category odds—and the interaction parameter of the ordinal MRF. The interaction parameter thus indicates the change in the logarithm of the adjacent category odds ratio. If 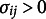 , the odds of observing one response in a higher adjacent category are greater than observing the other response in a higher adjacent category. If 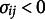 , the odds of observing one response in a higher neighboring category are smaller than observing the other response in a higher neighboring category.

In the derivations above, we already assumed that one of the category threshold parameters for each variable is zero. We inherited the identifying restriction that the threshold for the lowest category is zero, i.e., 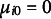 , from the GPCM. Let 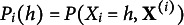 denote the probability that the variable *i* takes the value *h*. Then we can establish a relationship between the difference in category thresholds and the adjacent category odds: 

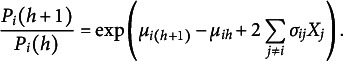

Note that this relation’s dependence on the interaction parameters is fine since we established their identification without using the category thresholds. We could fill in their log-odds expressions here.

Observe that we can only identify the difference in adjacent category threshold parameters. However, equipped with the restriction that the threshold for the lowest category is zero, i.e., 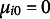 , we identify 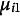 from 

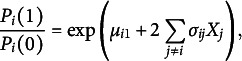

which we can then use to identify the other category thresholds. If there are no interactions between variables in the network (i.e., 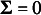 ), 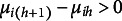 implies that there is a higher probability of observing responses in category 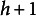 than in category *h*. Conversely, if 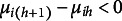 , there is a higher probability of observing responses in category *h* than in category 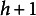 . In the face of network interactions, the term 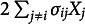 modifies these response tendencies. Note that this term is constant across neighboring categories. In this case, 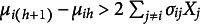 means that there is a higher probability of observing responses in category 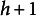 than in category *h*, and 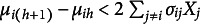 implies the opposite.

## 3 Bayesian edge selection

We now turn to the specification of the Bayesian model that will allow us to infer the structure of the MRF from empirical data. Bayesian variable or edge selection introduces binary indicator variables to model the inclusion of edges in the network: 

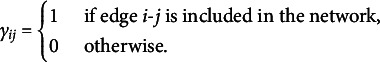

There are several ways to incorporate these indicator variables into the Bayesian model (Dellaportas et al., 2002; George & McCulloch, 1993; Kuo & Mallick, 1998; Tadesse & Vanucci, 2022). Marsman, Huth, et al. (2022) used a continuous spike and slab approach, imposing the following two-component mixture distribution as a prior on the interaction parameters of the Ising model 

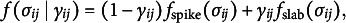

for 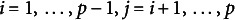 , where the spike distribution 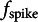 is concentrated around 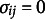 and the slab distribution 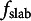 is a diffuse, continuous distribution also centered around zero. Here, 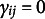 suggests removing the edge *i*–*j* from the network, since the prior on 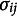 shrinks it to a negligible value close to zero. In contrast, 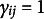 includes the edge and leads to a diffuse prior on 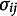 .

This article adopts a discrete spike and slab approach instead, using the following two-component mixture distribution as a prior on the interaction parameters (Gottardo & Raftery, 2008) 

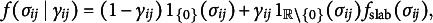

where 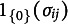 is an indicator function (i.e., a Dirac measure) that is equal to one when 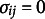 , and zero otherwise, and 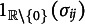 is an indicator function for the complementary event 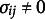 . Here, 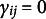 excludes edge *i*–*j* from the network since the prior on 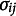 is a point mass at zero.

Specifying the spike distribution for the continuous spike and slab prior is nontrivial. For example, Marsman, Huth, et al. (2022) showed that arbitrarily setting the variance of the spike component can lead to an inconsistent structure selection routine (see also, Ly & Wagenmakers, 2022; Narisetty, 2022; Narisetty & He, 2014). The advantage of the discrete spike and slab prior over the continuous prior is that it is easier to formulate, since we do not need to specify the spike distribution. However, the disadvantage of the discrete spike and slab prior is that posterior inference using the Gibbs sampler becomes more complicated than for the continuous spike and slab prior. We address this issue in the next section.

In our implementation in bgms, we consider two choices for the slab component. The first is a Cauchy distribution, a common choice in Bayesian variable selection because it is diffuse and has heavy tails. We consider two scale values for the Cauchy: a scale of 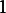 , which results in a 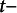 distribution with one degree of freedom, and a scale of 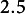 , which Gelman et al. (2008) suggested for logistic regression (albeit for centered predictors). The second choice for the slab component is a normal distribution with precision equal to the Fisher information matrix 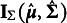 , which roughly gives the information about the 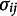 in a single observation (Kass & Wasserman, 1995; Raftery, 1999; Wagenmakers, 2007). We follow the approach of Ntzoufras (2009), who obtained good results by setting the off-diagonal elements of the covariance matrix to zero to make the spike and slab prior densities independent.^3^ Marsman, Huth, et al. (2022) also used this setup for the slab distribution in their continuous spike and slab approach to learning the structure of Ising models. Sekulovski et al. (2024) study the influence of different slab distributions on edge selection. We use the Cauchy distribution in our numerical illustrations and consider both unit information and Cauchy priors in the empirical example.

Specifying a prior distribution for the edge indicator variables completes the spike and slab prior setup. Throughout, we assume *a priori* that all MRF structures are equally likely, which implies that the edge indicators are independent, with prior inclusion probabilities equal to 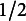 . This is a common choice for the prior inclusion probabilities in Bayesian graphical modeling (e.g., Marsman, Huth, et al. 2022; Mohammadi & Wit, 2019; Williams & Mulder, 2020). However, other choices are available (for an overview, see Section 3.6 in Consonni et al., 2018). Scott and Berger (2010) reported a hierarchical generalization of the standard Bernoulli setup that imposes a uniform prior density on the inclusion probability to account for model complexity. Marsman, Huth, et al. (2022) adopted this hierarchical specification. Both prior specifications are implemented in bgms, and Sekulovski et al. (2024) study their influence on edge selection.

To complete our Bayesian model, we specify independent beta-prime 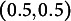 distributions on the threshold parameters 
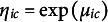
 , for 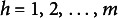 , 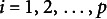 .

## 4 Markov Chain Monte Carlo edge selection

We are primarily interested in the posterior distribution of the vector of binary indicator variables 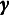 , i.e., the network structures. Ideally, we would have a conjugate prior for the threshold and interaction parameters, so that we could integrate them out and focus our attention on (e.g., Madigan & York, 1995) 

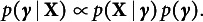

Unfortunately, such a conjugate prior does not exist, and we have to work with the joint posterior. 

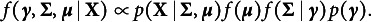

This joint posterior is intractable. Because of the discontinuity in the prior distribution of the interaction effects, we cannot use the expectation-maximization algorithm (Dempster et al., 1977) approach to variable or edge selection proposed by Ročková and George (2014). Therefore, we need to use a Markov chain Monte Carlo (MCMC) approach (e.g., a Gibbs sampler) to explore the joint posterior distribution by simulation (George & McCulloch, 1993).

The Gibbs sampler is the standard solution for sampling values from an intractable posterior distribution, but its application here faces two serious complications. The first challenge is that the target posterior distribution is doubly intractable (Murray et al., 2012) because, as will be explained below, the likelihood of the ordinal MRF is itself computationally intractable. The second challenge is that the discontinuity in the prior distribution for the interactions leads to a serious complication for the Gibbs sampler. We first address the challenge of the computational intractability of the likelihood of the ordinal MRF and then propose a straightforward transdimensional MCMC method that works for Bayesian models with the discrete spike and slab priors we use.

### 4.1 The computationally intractable likelihood

A key problem in the analysis of multivariate categorical models is that their normalizing constant tends to be computationally intractable. For example, for 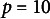 variables and 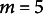 ordinal categories, the normalizing constant 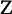 in Eq. (3) would consist of 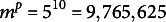 terms, which we would have to evaluate repeatedly in each iteration of the proposed Gibbs sampler. This is clearly computationally infeasible.

In network psychometrics, the problem of an intractable likelihood is usually solved by adopting the pseudolikelihood approximation proposed by Besag (1975), which replaces the intractable likelihood with a tractable one. This approximation underlies most existing frequentist methods for regularized parameter estimation of networks of discrete variables (e.g., Haslbeck & Waldorp, 2020; van Borkulo et al., 2014), but more recently pseudolikelihoods have also been incorporated into Bayesian methods (Marsman, Huth, et al. 2022; Mohammadi et al., 2023; Pensar et al., 2017). However, in other fields, especially in the area of social network analysis, several MCMC methods have been proposed that avoid the need to evaluate the full likelihood and its normalizing constant. Park and Haran (2018) provide a recent review of available approaches and show that the double Metropolis hastings (DMH) algorithm of Liang (2010) performs best overall in terms of effective number of samples from the posterior per second. In this article, we consider the pseudolikelihood approach but discuss the DMH algorithm and its implementation for Bayesian edge selection in Appendix C.

#### 4.1.1 The pseudolikelihood approach

The pseudolikelihood approach approximates the joint distribution of the vector variable 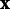 —i.e., the full MRF in Eq. (2)—with its respective full-conditional distributions (see Eq. (4)): 

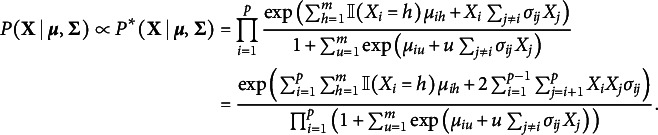

Note that the pseudolikelihood is equivalent to the full likelihood in Eq. (2), except that it replaces the intractable normalization constant with a tractable one. For the ten-variable network we considered earlier, the pseudolikelihood normalization constant has only 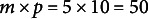 terms instead of the 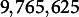 terms in case of the full likelihood.

Although the pseudolikelihood is a crude approximation to the full likelihood, the maximum pseudolikelihood estimates are consistent (Arnold & Strauss, 1991; Geys et al., 2007), and this is also the case for the pseudoposterior distribution (Miller, 2021). For exponential random graph models, a random graph model used in social network analysis, van Duijn et al. (2009) showed that pseudolikelihood estimates are generally biased in scenarios where there is only one observation of the network. For the Ising model, a graphical model used in network psychometrics, Keetelaar et al. (2024) showed that the bias in the pseudolikelihood estimates for the Ising model is comparable to that of the MLE in several scenarios typical of psychological applications. In these applications, we typically have many observations of the network. The overall good performance of the pseudolikelihood established by Keetelaar et al. (2024) applies especially to the joint pseudolikelihood approximation, the approximation we consider here, and less so to the disjoint pseudolikelihood approximation, also known as *nodewise regression*. Estimates based on the disjoint pseudolikelihood approximation can be severely biased, especially when the sample size is small. To verify that Keetelaar et al.’s results for the Ising model extend to our ordinal MRF, we examine the bias of the pseudolikelihood estimates of the parameters of the ordinal MRF relative to their maximum likelihood estimates in Appendix B and confirm that the bias of the two estimators is indeed comparable.

But there is no free lunch for the pseudolikelihood approach. Although it is fast, consistent, and also accurate in settings encountered in psychological applications, its standard errors can be underestimated. This was demonstrated by Keetelaar et al. (2024) for the Ising model. Despite this limitation, the pseudolikelihood approach can consistently estimate the unknown graph structure. Several studies on graph recovery using pseudolikelihood have established its consistency in high-dimensional settings where both *n* and *p* are allowed to grow (e.g., Barber & Drton, 2015; Meinshausen & Bühlmann, 2006; Ravikumar et al., 2010). Csiszár and Talata (2006) proposed an extension of BIC for pseudolikelihoods and showed that it can consistently reveal the Markov structure of the generating MRF in cases where the graphs grow in size but nodes have a finite number of neighbors. Pensar et al. (2017) used PIC to show that, under very general conditions, the marginal pseudolikelihood can consistently reveal the Markov cover of each node (the set of all neighbors of the node) and the global graph structure as the sample size increases (see also Vogels et al., in press, for a practical illustration).

### 4.2 A metropolis within Gibbs approach to Bayesian edge selection

The Gibbs sampler is the standard solution for sampling values from an intractable posterior distribution. As indicated above, the discontinuity in the prior distribution for the interactions creates a serious complication for the Gibbs sampler. To explain why this is the case, suppose we use the following block updating scheme: 

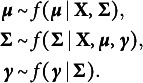

If we use this scheme and simulate the value 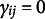 , we have to set 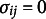 . But then we cannot move to other states, since 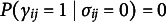 , and so 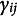 and 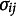 would have to remain at zero. The Markov chain is then said to be reducible (e.g., Norris, 1998).

As the Markov chain moves between two structures, we either exclude edges from the network and set their nonzero weights to zero, or include edges in the network and set the corresponding zero-value weights to nonzero values. As a result of these moves between models, the parameter dimensions change, and the MCMC method must account for these changes in order to generate a proper, irreducible Markov chain. Transdimensional MCMC methods have been proposed in the literature to account for these changes (e.g., Carlin & Chib, 1995; Green, 1995; Sisson, 2005). The most popular transdimensional MCMC method is the reversible jump algorithm (Fan & Sisson, 2011; Green, 1995), a Metropolis algorithm that uses auxiliary variables to adjust parameter dimensions as it moves between models. However, the reversible jump algorithm is difficult to tune and sensitive to how we adjust the parameter dimensions. Gottardo and Raftery (2008) viewed the discrete spike and slab as a mixture of mutually singular (MoMS) distributions—probability distributions that have disjoint support (see also Petris & Tardella, 2003, for earlier ideas)—and showed that reversible jump is similar to a Metropolis algorithm that jointly updates the indicator variable and the focal parameter (here 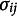 ). The advantage of this approach is that there is no need to specify a method for dimension matching as in reversible jump, and we can update the pairs 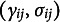 using the Metropolis algorithm. We embed the Metropolis pair update approach into the Gibbs sampler.

The proposed Gibbs sampler comprises two blocks of parameters to update 

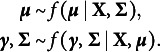

Although updating all parameters in a block at once is preferable because it minimizes autocorrelation, we choose to update the category thresholds one at a time and investigate two different strategies for updating the 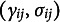 pairs, also one pair at a time. For the category thresholds, we do this because we can formulate an efficient sampler for the individual parameters. For the 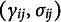 pairs, we do this because the transition kernels for multi-pair updates are difficult to design and implement.

Next, we discuss the proposed MoMS Gibbs sampler based on the pseudolikelihood approximation, called PL-MoMS, which is included in the R package bgms (Marsman et al., 2023) available from CRAN: https://CRAN.R-project.org/package=bgms. Appendix D discusses the proposed MoMS Gibbs sampler based on the DMH algorithm of Liang (2010), called DMH-MoMS, which is implemented in the R package dmhBGM (Marsman et al., 2024), which is available on Github: https://github.com/MaartenMarsman/dmhBGM.

#### 4.2.1 The PL-MoMS Gibbs sampler

**Block I: Updating the category thresholds.** To update the category thresholds, we need to sample from conditional distributions of the form 

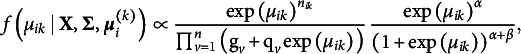

for 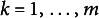 , and 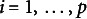 . Here, we have used 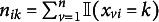 , 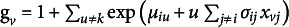 , and 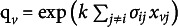 . These full conditionals are intractable, but note that their form resembles that of a generalized beta-prime distribution 

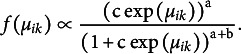

We will use the generalized beta-prime as a proposal in an independence chain Metropolis algorithm (Tierney, 1994). Maris et al. (2015) suggested using the properties of the target distribution to set the parameters of this proposal. This idea has also been successfully used by Marsman, Huth, et al. (2022) and Marsman and Huth (2023) to estimate the threshold parameters of the Ising and Divide and Color model (Häggström, 2001), respectively.

The log of the target distribution is concave and has linear tails: 

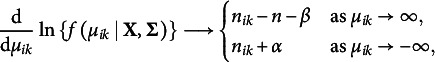

and the same holds for the proposal distribution: 

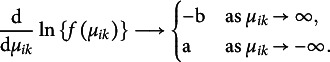

We match the tails of the proposal distribution to that of the target distribution by setting 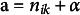 and 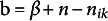 . The last free parameter, 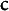 , is used to ensure that the proposal closely matches the target distribution at the current state of the Markov chain. Specifically, 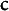 is used to make the derivatives of the logarithms of the proposal and target distributions equal. If 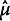 is the current state of 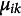 in the Markov chain, then we equate the two derivatives and solve *c*, which yields 

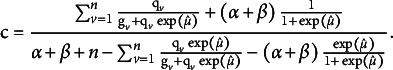

Now that we have the value for 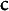 , we can sample a proposal from the generalized beta-prime distribution in the following way: we sample *Y* from a Beta 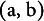 distribution and set the proposed value 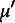 equal to 
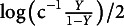
 . Here, 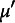 is a sample from the generalized beta-prime distribution. We accept the proposed value with probability 

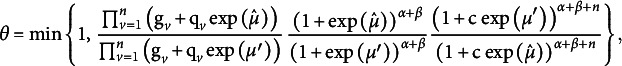

and retain the current value 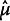 otherwise.

**Block II: Updating the edge indicators and interactions.** We base the proposal for Metropolis on the current state of the edge and interaction (i.e., 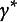 and 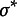 ), and we factor our proposal as 

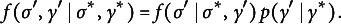

We will also use the MoMS formulation for the two proposal distributions. First, we consider the proposal distribution for the edge indicator, 

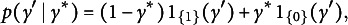

which proposes to change the edge, i.e., it sets 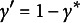 to make the between model move. Next, we define the conditional proposal distribution for the interaction effect, 

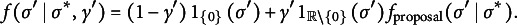

Here, the proposed value 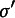 is equal to 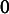 if 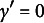 , and otherwise it is drawn from a proposal density 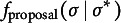 otherwise. We use a normal proposal distribution centered on the current state (i.e., a random walk) with variance 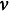 . We need to specify this variance parameter. We follow Kolovsky and Vanucci (2020) and Wadsworth et al. (2017) and use adaptive Metropolis to learn the variance 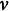 from the past performance of the Markov chain.^4^ We are now ready to formulate our Metropolis–Hastings approach.

Let 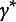 and 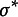 denote the current state of the edge indicator and the interaction between a variable *i* and *j*, and let 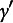 and 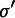 denote its proposed state. We accept the proposed states with probability 

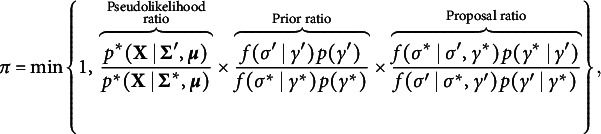

where 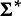 is the current state of the pairwise interaction matrix, and 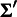 is the proposed state, with elements in row *i*, column *j*, and row *j*, column *i*, set equal to the proposed value 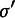 . If 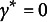 , we propose 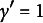 and 
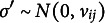
 and accept the proposed values with probability 

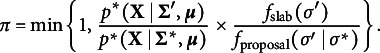

Conversely, if 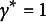 , we propose 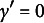 and 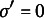 . We accept 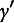 and 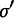 with probability 

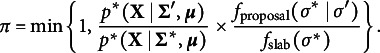

The pseudolikelihood ratios in the expressions above boil down to 

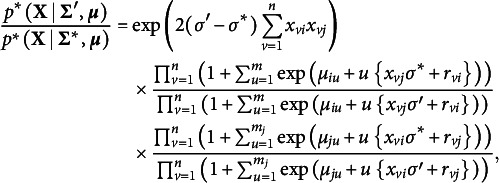

where 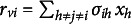 and 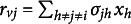 .

Two implementations of MoMS Metropolis have been reported in the literature. The Gibbs scheme in Wadsworth et al. (2017) applies MoMS Metropolis to each indicator-parameter pair in turn, and the add-delete scheme in Kolovsky and Vanucci (2020) applies it to a randomly selected indicator-parameter pair. The add-delete scheme adds an additional update of the “active” interactions (i.e., we update the 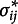 for which 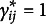 ) to speed up convergence (Gottardo & Raftery, 2008; Vanucci, 2022). Our approach is a hybrid of the two MoMS Metropolis approaches; we first consider a Gibbs scheme in which we update each indicator-parameter pair in turn, and then do an additional update of the active interaction parameters. Here we use a random walk Metropolis, i.e. we propose 
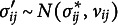
 . This scheme is implemented in the bgms package and is the one used in the numerical and empirical illustrations in the next two sections.

## 5 Numerical illustrations

In this section, we analyze the edge selection performance of PL-MoMS compared to DMH-MoMS. While the pseudolikelihood and the exact likelihood do not appear to lead to substantially different estimates (cf. the simulations in Appendix B), i.e. are unbiased, the pseudolikelihood may lead to smaller standard errors, or similarly, a reduced sensitivity to prior distributions. Since edge selection is modeled by the prior distribution on the pairwise interaction parameters, we examine how this affects the estimates of the statistical evidence for edge inclusion or exclusion for PL-MoMS and DMH-MoMS in Section 5.1. We then compare the performance of PL-MoMS and DMH-MoMS with two existing edge selection approaches for the binary MRF (i.e., the Ising model) in Section 5.2. Finally, we demonstrate the performance of our methods on ordinal data generated from the ordinal MRF in Section 5.3.

The R scripts and output needed to reproduce the numerical experiments and results shown in this section are available in an online repository at https://osf.io/qsj4w/. Our simulations were performed on the Dutch national supercomputer Snellius, but we estimate the runtimes of the proposed procedures on a MacBook Pro with an M1 Pro chip.

### 5.1 A comparison of evidence estimates

The pseudolikelihood-based estimates in Appendix B appear to have smaller variance than the stochastic estimates based on the exact likelihood, which could make the posterior distributions based on a pseudolikelihood less sensitive to the prior distribution than posterior distributions based on the exact likelihood. However, in our Bayesian edge selection procedure, the statistical evidence for the inclusion or exclusion of individual edges in the network is modeled by the spike and slab prior distributions on the pairwise interaction parameters. The reduced sensitivity to the prior distribution is likely to affect the estimated evidence for the inclusion or exclusion of edges in the network. Before analyzing the quality of our Bayesian edge selection procedure in the next two sections, we compare here how PL-MoMS-PL and DMH-MoMS estimate the statistical evidence.

In Bayesian edge selection, the evidence for the inclusion or exclusion of individual links in the network is measured by the inclusion probability. The posterior inclusion probability is the model-averaged probability of including an edge in the network, given the data 

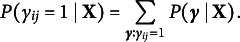

It indicates the probability that the edge is included in the network that generated the observed data and is essential for Bayesian analysis of graphical models. The edge inclusion probability is used to define the median probability structure—a single, optimal structure for predicting new observations (Barbieri & Berger, 2004)—and the inclusion Bayes factor. The inclusion Bayes factor is the change from the prior inclusion odds to the posterior inclusion odds, 

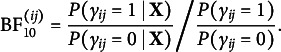

It indicates how much more likely it is that the observed data came from a network that included the edge *i*–*j* than from one that did not. The exclusion Bayes factor 
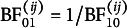
 gives the evidence for the conditional independence between nodes *i* and *j* in the data. Sekulovski et al. (in press) show that the inclusion Bayes factor, as a test for conditional dependence or independence of pairs of variables in the network, is robust to assumptions about the structure of the rest of the network. It also appears to be optimal for detecting evidence of conditional independence compared to Bayes factors that compare the same network structure with and without the link, or to credible interval-based tests.

We investigate how PL-MoMS and DMH-MoMS estimate the inclusion Bayes factors using simulated data, following the setup for the bias analysis in Appendix B, where we generated 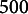 datasets, each with 300 observations ( 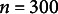 ) on 24 ordinal variables ( 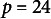 ) with five response categories ( 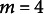 ). The datasets were generated from the ordinal MRF in Eq. (2). For each variable, four threshold parameters were sampled from a uniform distribution ranging from 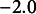 to 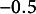 , sorted in decreasing order. The pairwise interaction parameters were sampled from a normal distribution with a mean of zero and a standard deviation of 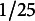 . For each of these 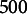 datasets, we estimate the joint posterior distribution of the model parameters and the edge inclusion indicators with PL-MoMS and DMH-MoMS, as described in Appendix D. It is generally recommended to run the *inner Gibbs sampler* of the DMH algorithm for 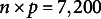 iterations, for each model parameter and edge indicator and pairwise interaction pair (i.e., 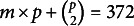 times) in each iteration of the Gibbs sampler. Clearly, this would make its application intractable, as confirmed by the runtime analysis in Appendix E. For this analysis, we therefore use a fixed number of 10 iterations for the inner Gibbs sampler. We ran PL-MoMS and DMH-MoMS for 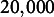 iterations for each of the 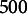 datasets. Analysis of a single dataset using PL-MoMS took about 5 min, and using DMH-MoMS with ten iterations of the inner Gibbs sampler took about 11 hr on a single core of a MacBook Pro with an M1 chip. We used the Snellius supercomputer to distribute the analysis of the 500 datasets in batches across 124 cores. On average, it took about 16 min to analyze a single dataset on a single core on Snellius with PL-MoMS and about 19 hr with DMH-MoMS.

**Figure 1:**
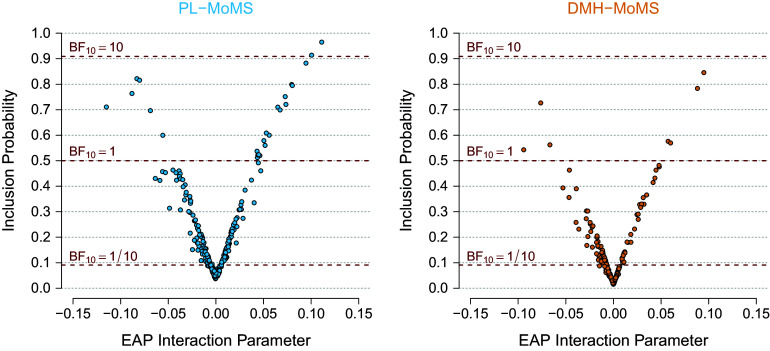
Scatterplots comparing EAP (expected a posteriori) estimates for the pairwise interaction parameter with the estimated inclusion probabilities, each averaged over 500 datasets. The left panel shows estimates based on the pseudolikelihood approach, while the right panel focuses on estimates based on the DMH algorithm. Each scatterplot includes dashed lines representing specific evidence thresholds under a uniform prior; the top line indicates an inclusion Bayes factor value of 10 (i.e., evidence for inclusion), the middle line indicates an inclusion Bayes factor value of 1 (i.e., absence of evidence), and the bottom line indicates an inclusion Bayes factor value of 0.1 (i.e., evidence for exclusion).

We computed the expected a posteriori (EAP) estimates or posterior means of the edge indicator variables (i.e., the inclusion probability) and the pairwise interaction parameters and averaged these estimates across the datasets. Figure 1 shows scatterplots of the averaged EAP estimates of the interaction parameters against the estimated inclusion probabilities, with estimates based on PL-MoMS in the left panel and those based on DMH-MoMS in the right panel. The gray dashed lines indicate the evidence thresholds corresponding to 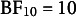 at the top, 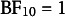 in the middle, and 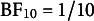 at the bottom. Note that the two scatterplots show a similar relationship between the interaction effects and the inclusion probabilities, but that PL-MoMS tends to show more evidence for edge inclusion and less evidence for edge exclusion than DMH-MoMS, and we therefore expect PL-MoMS to show higher sensitivity and lower specificity than DMH-MoMS. Note that the estimates for the pairwise interactions shrink to zero more for DMH-MoMS than for PL-MoMS, which is a consequence of the posterior distribution based on the pseudolikelihood being less influenced by the prior distribution. Because the EAP estimates based on the pseudolikelihood are further from zero, the pseudolikelihood approach results in increased sensitivity or decreased specificity.

### 5.2 Bayesian edge selection with binary data

Now that we have a better understanding of how PL-MoMS and DMH-MoMS accumulate evidence, we want to evaluate their impact on Bayesian edge selection. We also want to compare our proposed PL-MoMS and DMH-MoMS procedures with two alternative approaches for the Ising model, the EBIC-Lasso approach of van Borkulo et al. (2014) and the Bayesian edge selection method of Park et al. (2022), which uses a continuous spike and slab prior and the DMH algorithm to sample from the posterior distribution. We refer to the latter as DMH-CSaS (i.e., DMH combined with a *Continuous Spike and Slab*). Based on the results of Sekulovski et al. (in press) that we discussed earlier, we expect that our discrete spike and slab prior approach will be similar to EBIC-Lasso in that the method will have a high specificity or true negative rate and a low sensitivity or true positive rate. However, given what we learned about PL-MoMS and DMH-MoMS in the previous section, we expect this effect to be partially mitigated by the pseudolikelihood approach, and thus expect PL-MoMS to result in lower specificity and higher sensitivity than DMH-MoMS. Finally, given the above expectations and the results in Park et al. (2022), we expect PL-MoMS and DMH-MoMS to have higher specificity but possibly lower sensitivity than DMH-CSaS.

We wanted to compare our methods with the DMH-CSaS method proposed by Park et al. (2022), but because we were unable to use their software implementation, we replicated their simulation setup for a direct comparison. This involved generating 500 datasets from the Ising model (i.e., the ordinal MRF with 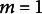 ), each with 24 variables and 300 observations. Each dataset was simulated with distinct parameter values: category threshold parameters were uniformly sampled between 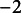 and 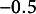 , and for the 276 edges, 69 were assumed present. The interaction parameters for these edges were sampled from a uniform distribution, with 41 of them sampled uniformly between 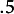 and 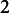 , and the remainder between 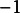 and 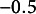 . Datasets containing variables with zero variance (67/500) were excluded from our analyses.

We estimated the joint posterior distribution of the model parameters and the edge inclusion indicators with PL-MoMS and DMH-MoMS. As in the previous simulations, the inner Gibbs sampler for DMH was run for ten iterations, and we ran our Gibbs samplers for 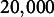 iterations on each of the 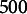 datasets. As before, the slab distribution is a Cauchy distribution with a scale of 2.5. We used the IsingFit R package (van Borkulo et al., 2016) with package defaults and the AND rule to estimate the parameters of the Ising model using EBIC-Lasso. Our results with IsingFit are almost identical to those reported by Park et al. (2022), confirming that our results should be comparable.

We estimated the median probability model using the two MoMS Gibbs samplers, which includes an edge in the network if its corresponding inclusion probability exceeds .5, and excludes it from the network otherwise. For EBIC-Lasso, we selected the edges that had a nonzero estimate and excluded the others. For each method, we compare how well they were able to recover the generating network structure by computing its specificity or true negative rate —TN / (TN + FP)—, sensitivity or true positive rate —TP / (TP + FN)—, and the Rand index (Rand, 1971) —(TN + TP) / (TN + FP + TP + FN). The results are in Table 1; we also copied the original results from Park et al. (2022).

**Table 1:** Performance metrics—specificity, sensitivity, and Rand index—of different edge selection methods applied to 500 simulated Ising model data sets, following the setup of Park et al. (2022)

Measure	PL-MoMS	DMH 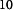 -MoMS	DMH 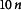 -CSaS 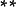	EBIC-Lasso
Specificity	0.849	0.949	0.786	0.997
Sensitivity	0.664	0.541	0.738	0.218
Rand index	0.802	0.847	0.774	0.803

*Note*: It includes the proposed PL-MoMS and DMH-MoMS approaches, the 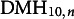 -CSaS approach of Park et al. (2022) ( 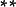 results copied from their Table 1), and the EBIC-Lasso method of van Borkulo et al. (2014).

Table 1 shows important differences in performance between the Bayesian edge selection methods and EBIC-Lasso. EBIC-Lasso performed best at identifying which edges were missing from the generating structures, i.e., has a high specificity, while DMH-CSaS performed worst at this task. DMH-MoMS was close to EBIC-Lasso on this metric, while PL-MoMS performed significantly worse. Conversely, DMH-CSaS performed best at identifying which edges were present in the generating structures, i.e., has a high sensitivity, while EBIC-Lasso performed worst on this metric. Interestingly, the DMH-MoMS method was not close to EBIC-Lasso on this metric, as it did much better, but it did not perform as well as PL-MoMS on this metric. These effects were as expected.

The methods thus trade off their specificity and sensitivity differently. Methods that perform well in terms of specificity tend to perform poorly in terms of sensitivity, and vice versa. We used the Rand index to measure how these trade-offs occur and to characterize the overall performance of the methods in recovering the generating network structure. The DMH-MoMS Gibbs sampler performed best on this overall metric with 85% correct identifications, while DMH-CSaS performed worse with 77% correct identifications. Interestingly, although EBIC-Lasso and PL-MoMS trade off specificity and sensitivity differently, they perform quite similarly on this metric with 80% correct identifications.

### 5.3 Bayesian edge selection with ordinal data

We have seen that PL-MoMS and DMH-MoMS are very capable of recovering the underlying network structure when applied to binary data. In the next analysis, we will see if this good performance generalizes to the analysis of ordinal data with 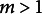 . Ideally, we would like to keep the simulation setup for 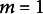 and 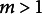 as similar as possible to render results comparable. However, there are two complications with this. First, the parameters of the Ising model and the ordinal MRF are not standardized, and the generating parameter values we used for the Ising model lead to degenerate versions of the ordinal MRF. Like the Ising model, when the parameters of the ordinal MRF exceed certain critical thresholds, the model exhibits a phase transition and from that point on produces data with zero variance. Unfortunately, it is generally unknown what these critical thresholds are. We address this by rescaling the parameter values. This brings us to the second complication. Our Bayesian edge selection procedure imposes a fixed spike and slab prior distribution on the pairwise interaction parameters; in our previous analysis, we used a Cauchy with a scale of 2.5 as the slab distribution, but this prior does not account for this rescaling of the interaction parameters. Because of this, and because we expect smaller interaction effects as the number of categories increases, we expect the two MoMS procedures to show increased specificity and decreased sensitivity, all else being equal.

We investigate the Bayesian edge selection performance of PL-MoMS and DMH-MoMS on 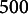 datasets, each with 300 observations ( 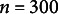 ) on 24 ordinal variables ( 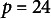 ) with five response categories ( 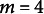 ) simulated from the ordinal MRF in Eq. (2) as follows. For each variable, four threshold parameters were sampled from a uniform distribution ranging from 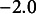 to 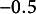 , sorted in decreasing order. As in the binary case, we assume that 69 out of 276 edges were present and the rest were absent. However, where the products 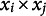 were zero or one in the binary case, they now range from zero to 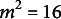 . To account for this change in value, we sampled the interaction parameters in the same way as before but multiplied them by a factor of 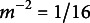 . Thus, the interaction parameters for the current edges were sampled from a uniform distribution, with 41 of them uniformly sampled between 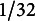 and 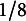 , and the rest between 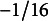 and 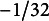 . There were no datasets containing variables with zero variance.

Similar to the analysis in the previous section, we estimated the joint posterior distribution of the model parameters and the edge inclusion indicators with PL-MoMS and DMH-MoMS. As before, the inner Gibbs sampler for DMH was run for ten iterations, and we ran our Gibbs samplers for 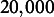 iterations on each of the 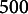 datasets. The slab distribution is again a Cauchy distribution with a scale of 2.5. We expect that using the same scale for the Cauchy slab distribution while shrinking the generating interaction parameters would lead to increased specificity and decreased sensitivity, a scaling effect that is essentially a form of the Jeffreys–Lindley paradox in Bayesian hypothesis testing (Jeffreys, 1961; Lindley, 1957). To investigate this, we estimate the joint posterior distribution using PL-MoMS with a rescaled Cauchy 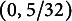 slab distribution. We did not do this for the DMH-MoMS approach because i) we think we can judge the effect of rescaling on this procedure from the effect of rescaling on the PL-MoMS approach, and ii) running the DMH-MoMS approach twice is quite time consuming even on a supercomputer (it took us about 2.5 days to analyze the 500 datasets on Snellius) and expensive.

Table 2 shows that, as expected, when we do not correct the scale of the slab distribution for the increased pairwise interaction effects, or for the fact that the parameters must now shrink in size, the specificity of the variable selection methods increases while their sensitivity decreases compared to the results in the binary case shown in Table 1. It becomes more difficult to detect the smaller effects because the prior is much more diffuse compared to the size of the effect in the binary case. Of the absent edges, PL-MoMS correctly identified about 85% in the previous analysis and now 97%. Similarly, the DMH-MoMS approach, which had 95% correct before, now has a near perfect score on this metric. However, as mentioned above, they performed less well in detecting the edges that now have very small edge weights. Where PL-MoMs correctly identified 66% of the present edges, this has now shrunk to 42%, and for DMH-MoMs it has shrunk from 54% to 20%. While the relative performance of PL-MoMs compared to DMH-MoMs is the same as before, in that DMH-MoMs has higher specificity and lower sensitivity, the fact that DMH-MoMs has such low sensitivity now makes its overall performance of 80% less than PL-MoMs’ 83%.

**Table 2:** Performance metrics—specificity, sensitivity, and Rand index—of the PL-MoMS and the DMH-MoMS methods applied to 500 simulated ordinal MRF datasets

Measure	PL-MoMS	DMH 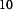 -MoMS	PL-MoMS w. scale 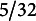
Specificity	0.969	0.996	0.849
Sensitivity	0.418	0.204	0.644
Rand index	0.832	0.798	0.797

We also performed an analysis with PL-MoMS correcting for the rescaling of the size of the pairwise interactions. This correction worked perfectly in that, despite the additional parameters that need to be estimated for the ordinal model, it shows almost identical performance on all three metrics as before. The specificity was 85% in both scenarios, the sensitivity decreased slightly from 66% to 64%, and the overall performance of 80% was also the same in both scenarios. This result suggests that correcting for the number of categories in the scale of the slab distribution can effectively mitigate differences in the size of the interaction effects.

## 6 A Bayesian reanalysis of McNally et al. (2015): A network approach to posttraumatic stress disorder

To illustrate the value of the proposed Bayesian methodology in applied research, we reanalyze data on posttraumatic stress disorder (PTSD) symptoms from McNally et al. (2015). Specifically, we compare the Bayesian estimate of the network using PL-MoMS with those obtained in the original study using an unregularized frequentist GGM and the now popular regularized variant. In addition, we use the new Bayesian approach to analyze the available evidence for conditional independence and conditional dependence, which is not possible with the Frequentist approach. We also use the reanalysis to discuss practical issues such as prior robustness and compare the inclusion Bayes factors under different prior specifications. Our online repository at https://osf.io/qsj4w/ includes a fully reproducible R tutorial on using bgms that reproduces the analysis below.

McNally et al. (2015) reported a network analysis of PTSD symptoms of 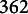 Chinese adults who survived the Wenchuan earthquake and lost a child in the disaster. McNally et al. (2015) used the 17 items from the Mandarin Chinese version of the Posttraumatic Checklist (civilian version), each of which assesses a symptom of PTSD according to the DSM-IV (American Psychiatric Association, 2000). Participants rated how much the symptom bothered them in the past month on a 5-point scale from 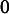 (not at all) to 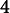 (extremely). We excluded 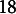 participants from the analysis because they had one or more missing responses.

**Figure 2:**
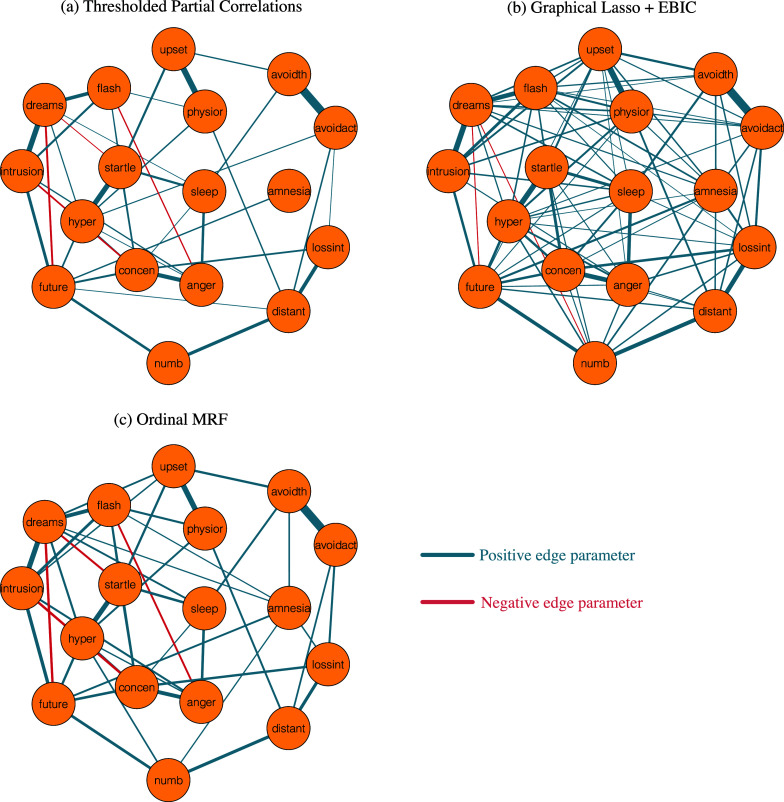
Markov random fields estimated with different approaches. Panel (a) displays the network structure as reported in McNally et al. (2015) which thresholded observed partial correlations at 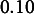 ; panel (b) shows the GGM estimated with the popular graphical Lasso + EBIC approach; and panel (c) displays the ordinal MRF estimated with the Bayesian approach presented in this paper.

The original analyses treated the observed ordinal responses as continuous variables and analyzed the network using partial correlations. No regularization or model selection procedures were available when the original paper was published, so a cutoff simply suppressed partial correlations below 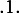 Figure 2(a) shows this network. Recent guidelines (Isvoranu & Epskamp, 2023) suggest using graphical lasso regularization (glasso; Friedman et al., 2008) in combination with EBIC model selection (Chen & Chen, 2008). This network is shown in Figure 2(b). We can see that the two networks are very different. For example, the partial correlation network which uses a cutoff for the partial correlations is much sparser than the EBICglasso network. The sparsity of the partial correlation network is not only due to the use of a cutoff on the partial correlations, since some of the relations in the partial correlation network are missing in the EBICglasso network. This brings us to a second difference between the two networks. While several negative interactions were found in the thresholded partial correlation network (e.g., “intrusion”–“hyper”), some were removed (e.g., “dreams”–“startle”) or differently weighted in the EBICglasso network (e.g., “dreams”–“future”).

### 6.1 Structure estimation in the presence of uncertainty

The two classical methods each provide a single network estimate but do not quantify how certain we are about this structure. In contrast, our Bayesian variable selection approach in principle provides the uncertainty (i.e., posterior probability) associated with each possible structure. In this setting, the model or structure with the highest posterior probability is then often selected. Under certain prior specifications, choosing the model with the highest posterior probability is, in principle, the estimate provided by classical methods (see Marsman, Huth, et al. (2022), for a brief discussion). However, selecting the model with the highest posterior probability can be problematic for several reasons. First, we must estimate the posterior structure probabilities, and since we have no analytic expression for their values and there are too many possible structures to enumerate, we are often uncertain about which structure has the highest posterior probability, especially if we are uncertain about the underlying structure. Second, even if we were certain about their exact values, it is often the case that the most likely structure is not that much more plausible than the second most likely structure. To address this latter issue, Barbieri and Berger (2004) suggest using the median probability model, as it has an optimal predictive value even in the face of model uncertainty. The median probability model selects only those variables (edges) with a posterior inclusion probability greater than 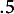 .

To measure our uncertainty about the underlying structure for the problem at hand, we tracked how many different structures the MCMC procedure visited. Assuming the unit information prior for the interaction effects, we ran the MCMC procedure for one million iterations, which visited 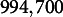 different structures. The most likely structure accounted for less than 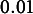 percent (one hundredth of one percent) of the posterior probability. These results show that we are very uncertain about the structure of the network; many structures seem plausible for the data at hand, and no structure stands out. We therefore consider the median probability model shown in Figure 2(c). Note that the median probability model is more densely connected than the thresholded partial correlation network, but unlike the EBICglasso network, it retains the negative associations.

### 6.2 Edge and structural evidence

Importantly, the fact that we are massively uncertain about which overall network structure is correct does not mean that we cannot be highly certain about specific edges. For example, while a number of edges may be present in some structures and not in others, leading to high uncertainty about the overall structure, other edges may be present (or absent) in almost all structures, and we can therefore be highly certain about their presence (or absence). A major advantage of Bayesian model averaging is that it allows us to express this uncertainty locally in terms of the Bayes factors for edge inclusion. Can we interpret the missing edge as evidence of conditional independence, or should we be more cautious with this interpretation?

**Figure 3:**
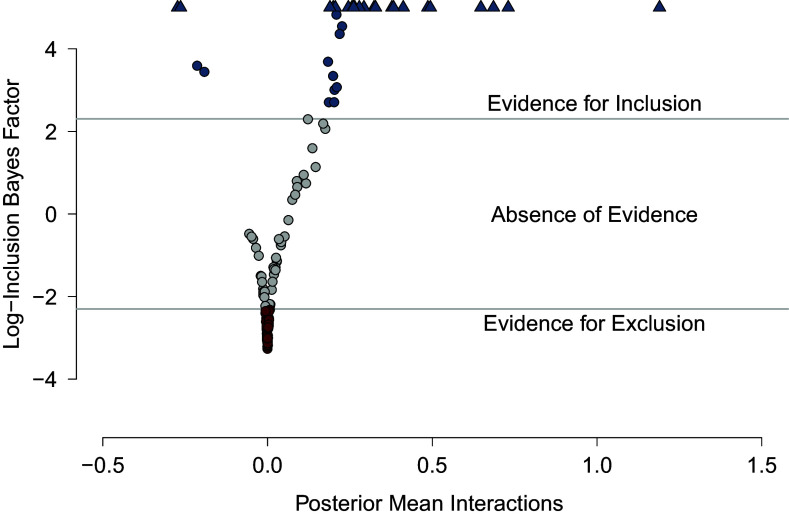
The mean of the model-averaged posterior distribution of the interaction parameters (x-axis) plotted against the log of the corresponding inclusion Bayes factor (y-axis). Positive values of the log Bayes factors indicate evidence for inclusion, and negative values indicate evidence for exclusion. We use Bayes factor values between 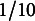 and 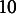 to indicate weak (or no) evidence. For this analysis, we assumed a unit information prior on the interaction parameters. Triangles indicate log Bayes factors greater than five.

**Figure 4:**
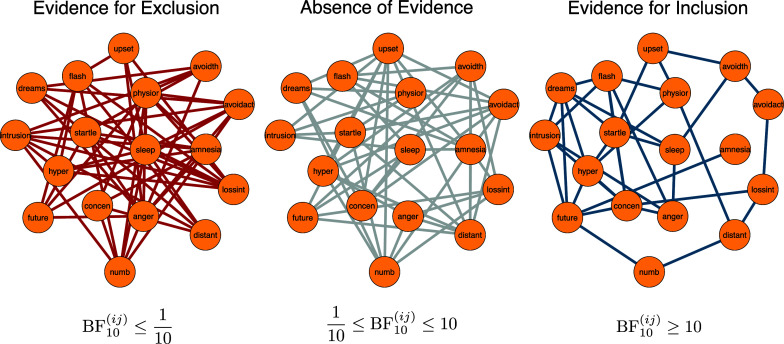
Three networks showing the level of evidence for each edge. In the network on the left, the edges reflect inclusion Bayes factors less than 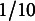 ; in the middle network, the edges reflect Bayes factors between 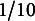 and 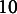 ; the edges in the network on the right reflect Bayes factors greater than 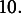 For this analysis, we specified a unit information prior on the interaction parameters.

The Bayesian methodology allows us to answer this question in a straightforward way. Figure 3 shows a scatterplot of the model-averaged posterior means of the interaction parameters (x-axis) against the logarithm of the corresponding inclusion Bayes factor (y-axis). This illustrates how the value of the Bayes factor increases as the corresponding posterior distribution moves away from zero. In the figure, we interpret an inclusion Bayes factor less than 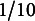 as evidence of edge exclusion, i.e., conditional independence. These inclusion Bayes factors are colored red. Similarly, we interpret inclusion Bayes factors greater than 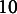 as evidence of edge inclusion, i.e., conditional dependence. These inclusion Bayes factors are colored in blue. The blue triangles or arrows at the top indicate the inclusion Bayes factors that we estimate to be greater than 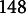 . This means that we can be confident that these edges are present in the data generating model. On the other hand, for the many grey edges indicating *absence of evidence*, we likely would not want to draw strong conclusions about their presence or absence in the data-generating model. Another way to present these results is with the three network plots in Figure 4, which show for which relations there is *evidence of absence* (left panel), *absence of evidence* (middle panel), and *evidence of presence* (right panel).

The inclusion Bayes factors convey the evidence for individual edges. But they can also help us identify parts of the network about which we are confident and parts about which we are uncertain. For example, the right panel of Figure 4 shows that we have conclusive evidence for each of the edges between the symptoms “flashbacks,” “dreams,” and “intrusions.” Let 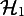 denote the hypothesis that the three variables form a clique (i.e., there is an edge between each variable), and let 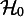 denote its complement (i.e., at least one of the edges is missing). The Bayes factor in which we pit the two hypotheses against each other is equal to 

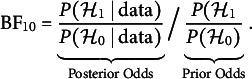

There are eight different configurations of edges between the three variables, one corresponding to 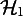 and seven corresponding to 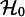 . Since all structures are equally likely *a priori*, the prior probability is 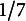 in favor of 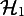 . Our estimate for the posterior probability that the data come from a network in which the three variables form a clique is close to one, i.e., 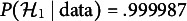 , and the estimated posterior odds in favor of 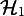 are 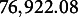 . The Bayes factor is thus estimated to be 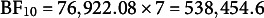 , indicating massive evidence in favor of 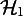 . In contrast, we have inconclusive evidence between the symptoms “avoidth” (avoidance of thinking about a stressful experience), “amnesia,” and “flashbacks.” We saw all 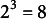 possible configurations of edges between these three variables, and no structure stood out.

### 6.3 Prior robustness

The qualitative conclusions drawn from the Bayesian analysis of the ordinal MRF may be sensitive to the choice of the prior distribution. To assess the robustness of the results, we perform a robustness analysis comparing the results of the unit information prior to the Cauchy prior with different scale values. We report the details of this analysis in the Appendix F. Some of the conclusions of our analysis would change depending on the choice of a more or less diffuse prior, especially the BFs that are close to our cutoffs of 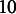 or 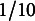 . At the same time, however, we found that many results were robust to changes in our prior specifications, especially the BFs that are far from our chosen cutoffs. Of the 52 BFs that showed compelling evidence for conditional independence, i.e. edge exclusion, using the unit information prior, 74 showed compelling evidence after adopting the more diffuse Cauchy 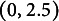 prior density. Thus, while for 22 edges we now conclude that we have *evidence of absence* rather than *absence of evidence*, for 52 edges we did not change our conclusions for either prior specification. Conversely, of the 35 BFs that showed compelling evidence for conditional dependence, i.e., edge inclusion, using the unit information prior, 32 showed compelling evidence after adopting the more diffuse Cauchy 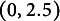 prior density. Thus, while we now conclude that we have *absence of evidence* rather than *evidence of presence* for three edges, we have not changed our conclusions for 32 edges under either prior specification. In sum, the unit information prior appears to be the more conservative choice in this analysis.

## 7 Discussion

We introduced a Markov random field (MRF) for ordinal variables based on existing work in the psychometric Anderson and Vermunt (2000) and machine learning literature Suggala et al. (2017). In addition, we propose a Bayesian variable selection method that allows one to model the inclusion and exclusion of individual edges in the network and, consequently, to test for conditional dependence and independence of network variables using the inclusion Bayes factor. We provide an implementation in the R-package bgms, which we have used to determine the performance of our methodology through simulation, and to analyze empirical data to illustrate how our methodology can be used in applied research. We conclude this article by discussing the relations of the proposed ordinal MRF to other multivariate models for ordinal data, commenting on how the prior distributions in the Bayesian model affect our tests for conditional dependence and independence of the network variables, our approach for analyzing the intractable likelihood, and suggesting future improvements of the implementation in bgms.

### 7.1 The ordinal MRF and other multivariate models for ordinal variables

We introduced the ordinal MRF as the marginal distribution of a latent variable model for ordinal variables—the GPCM—and linked its conditionals to the univariate adjacent category logit (e.g., Anderson & Vermunt, 2000; Suggala et al., 2017). One alternative to the proposed ordinal MRF is the multivariate probit model (e.g., Guo et al., 2015), which maps the observed categories of ordinal variables onto the adjacent intervals of Gaussian variables and models the latent Gaussian variables with a GGM. Another alternative model that uses an underlying continuous latent variable is the Gaussian copula graphical model (GCGM; Dobra & Lenkoski, 2011), which is based on the extended rank likelihood of Hoff (2007) and takes into account transformations of the underlying Gaussian variables. These models are much easier to analyze than the ordinal MRF, and an excellent Bayesian treatment of the GCGM is implemented in the R package BDgraph (R. Mohammadi & Wit, 2019). However, in contrast to the model proposed in this article, and although the underlying GGM is an MRF, the Markov properties do not hold in the marginal distribution of the ordinal variables for the multivariate probit or the GCGM (e.g., Dobra & Lenkoski, 2011; Liu et al., 2009).

The multivariate probit model and the GCGM have not been popular options for analyzing ordinal data in psychological research. In psychology, researchers often choose to use misspecified models, either by dichotomizing their ordinal data and analyzing the binarized data with an Ising model or by treating the ordinal data as continuous and analyzing it with a GGM. The use of misspecified models raises practical and theoretical concerns (e.g., Johal & Rhemtulla, 2023; Liddell & Kruschke, 2018). For example, the recovery of network structure using dichotomized data depends critically on the specific threshold for dichotomization (e.g., Hoffman et al., 2018), and the recovery of network structure by assuming continuity cannot be guaranteed (e.g., Loh & Wainwright, 2013). With the proposed ordinal MRF, researchers can consistently recover the underlying network structure of their ordinal data.

### 7.2 Prior specification for Bayesian edge selection

**Figure F1:**
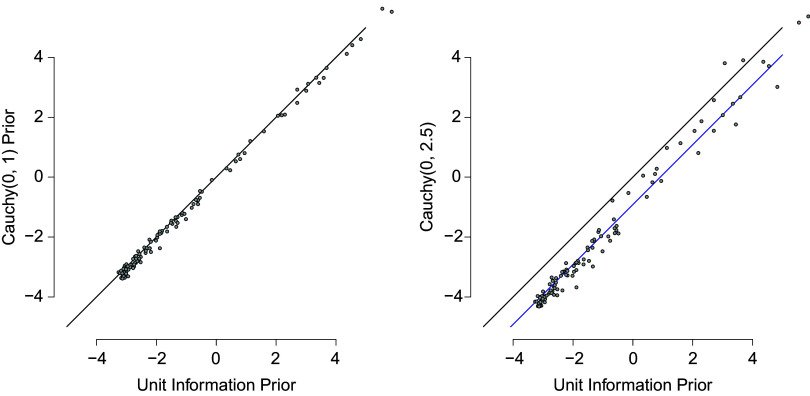
Scatterplots of the log of the inclusion Bayes factors under the unit information prior versus the log of the inclusion Bayes factors under the Cauchy 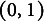 prior in the left panel and under the Cauchy 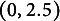 prior in the right panel. The black diagonal line passes through zero and has unit slope. The blue diagonal goes through 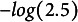 and also has unit slope.

We used Bayesian variable selection to model the selection of edges in the network structure, and the discrete spike and slab prior on edge weights is central to this approach. The discrete spike and slab prior uses a latent binary edge indicator to assign edge weights to a diffuse slab distribution to indicate edge presence, or it is set to zero to indicate edge absence. This setup requires us to choose a distribution for the diffuse slab component and for the latent edge indicator. We have chosen to focus here on the slab specification and have considered two types of distributions, the unit information prior and the Cauchy distribution. In our empirical example (e.g., Figure F1), we found that more diffuse prior distributions tend to lead to more evidence for the null hypothesis (i.e., edge exclusion). Here, the Cauchy distribution with a unit or larger scale was more diffuse than the unit information prior. This particular form of sensitivity to the prior distribution is well known in Bayesian hypothesis testing (Jeffreys, 1961; Lindley, 1957). More diffuse prior distributions tend to give increasing support to extreme parameter values that make predictions that the observed data cannot support. In contrast, the null hypothesis makes an exact, and thus risky, prediction that the observed data can partially support, even in the nonnull scenario. The relative evidence for the null hypothesis then increases as the support for the alternative hypothesis diminishes. While the prior specification clearly affects tests of the network structure, we paid relatively little attention to this aspect of the Bayesian model. Although we devoted little effort to its analysis, this aspect of our model is obviously important. See, for example, the expression for the inclusion Bayes factor. More work is needed to formulate good default specifications for the prior on the structure of psychological networks.

The partial association parameters of discrete variable MRFs are unstandardized effects, meaning that what constitutes a plausible range for their values depends very much on the size of the network and the number of response categories. The latter effect is of course new, as the ordinal MRF is new, and has been demonstrated in our numerical illustrations; increasing the number of response categories while fixing the scale of the prior on the pairwise interactions increases the evidence for the null effect hypothesis. This is essentially a form of the Jeffreys–Lindley paradox (Jeffreys, 1961; Lindley, 1957). These scaling effects pose a new and unresolved challenge for Bayesian analysis of MRFs for discrete variables, and in particular for setting a good standard for priors on partial associations. We have shown that reducing the scale of the prior in proportion to the increase in the range of the product in the pairwise interactions can effectively mitigate the effects of an increased number of response categories in our simulations. However, further research is needed to determine whether this strategy can effectively help to standardize the pairwise interaction effects across models for variables with different ranges of categories, and whether a similar strategy can be applied to standardize them across models with different numbers of variables. We leave this for future research.

### 7.3 Dealing with the intractable normalizing constant of the ordinal MRF

The normalization constant of the ordinal MRF poses a serious computational challenge to its analysis. We proposed and analyzed two statistical approaches that bypass its direct computation, the double Metropolis–Hastings (DMH) algorithm and the pseudolikelihood approach. Both approaches introduce an approximation to bypass the computation of the normalization constant of the ordinal MRF. The DMH algorithm applies an approximation to the transition kernel of the underlying Metropolis–Hastings algorithm, while the pseudolikelihood provides a coarser approximation that replaces the intractable likelihood with a tractable one. When properly implemented, the approximation used by the DMH algorithm allows us to closely approximate the full posterior distribution of the ordinal MRF, while inference based on the pseudolikelihood remains a cruder approximation (i.e., is known to underestimate the posterior variance; Miller, 2021).

The correctness of the approximation used by the DMH algorithm depends on the ability of its inner Gibbs sampler to approximate a draw from the full likelihood. This inner Gibbs sampler introduces a new computational challenge. While previous work suggested running the inner Gibbs sampler for a number of iterations equal to the dimension of the data at hand (e.g., Liang, 2010) or up to ten times the sample size (e.g., Park & Haran, 2018; Park et al., 2022), our runtime analysis showed that running the inner Gibbs sampler for this many iterations is not feasible. To make the use of the DMH algorithm remotely feasible in our analyses, we set the number of iterations of the inner Gibbs sampler to ten. Although feasible, the ten iterations for the inner Gibbs sampler were far fewer than the heuristic choices of 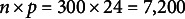 iterations based on data size or the 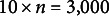 iterations based on sample size for our analyses. As a result, it is unclear how close the DMH approximation was to the full posterior distribution.

Computational cost aside, we expect that both our implementation of the DMH algorithm and the pseudolikelihood approach provide a somewhat crude approximation to the full posterior distribution. Nevertheless, we have shown that both approaches are able to recover the underlying graph structure, although they accumulate evidence differently and trade off specificity and sensitivity in different ways. Sensitivity was higher for the pseudolikelihood approach, which we attribute to the underestimation of the posterior variance by the pseudoposterior, while specificity was higher for the DMH approach. Overall, the DMH algorithm performed best in recovering the underlying graph structure in terms of the Rand index, although the performance of the pseudolikelihood approach was similar.

The pseudolikelihood approach was particularly effective in overcoming the computational challenge, while our implementation of the DMH algorithm with ten iterations of the inner Gibbs sampler exhibited significantly longer run times compared to the pseudolikelihood approach. This discrepancy raises practical considerations for method selection. Parallelization was shown to reduce the runtime of the DMH algorithm. However, this reduction was rather limited, and the practicality of this solution is also limited for applied researchers without access to advanced computing resources. Therefore, the feasibility of parallelization in increasing the computational speed of DMH needs further consideration.

Given the massive computational overhead of the DMH algorithm over the pseudolikelihood approach, and the relatively good graph recovery performance of the latter compared to the former, we recommend that researchers use the pseudolikelihood method. However, we believe that progress can be made to mitigate the problems introduced by approximating the likelihood with the pseudolikelihood approach. In the context of social network analysis, Bouranis et al. (2017, 2018) proposed to correct the mode and curvature around the mode of the pseudolikelihood using stochastic versions of maximum likelihood estimates (MLEs) and the Hessian matrix. However, as shown in Appendix B, which extends the results of Keetelaar et al. (2024) for the Ising model, we believe that we do not need to correct the mode of the pseudolikelihood using the MLEs, since their stochastic versions appear to agree with the maximum pseudolikelihood estimates. Therefore, we believe that future work should focus on correcting the curvature around the mode.

### 7.4 The practical benefits of Bayesian analysis of MRFs

The proposed Bayesian methodology allows us to model the uncertainty associated with the structure of the network and to express the relative plausibility of different structures for the data at hand. Our reanalysis of the PTSD data from McNally et al. (2015) allowed us to show that we can be very uncertain about which particular structure underlies our data. This is a big deal. If we see that we are uncertain about the structure of the network, we know that we should be cautious about using it to build theories, interventions, or policies. Conversely, if we are not aware of this uncertainty, we run the risk of being overconfident in our results (Hoeting et al., 1999). Because the methodology for assessing the uncertainty underlying our networks is new, researchers are unaware of this uncertainty. And since this uncertainty can be substantial in practice, as our empirical analysis has shown, we understand why researchers are concerned about the robustness of network results (Fried & Cramer, 2017; Jones et al., 2021).

Our reanalysis of the PTSD data from McNally et al. (2015) also showed that we can use our methods to determine that even if we are uncertain about the structure of the network, we can be confident about some of its substructures. We used Bayesian model averaging to aggregate what we know about individual structures to accumulate the evidence for edge inclusion or exclusion. We believe that the inclusion Bayes factor (BF)—which expresses the statistical evidence for edge inclusion—is a major step forward in the statistical analysis of psychometric networks. It provides a formal test of conditional independence that is insensitive to the specification of the rest of the network structure. While we proposed the inclusion BF to evaluate the support for individual edges, we also showed that we can extend it to a particular configuration of a subset of edges in the network. For example, in our reanalysis of the PTSD symptom data, we provided strong evidence that the data came from a network that included all edges between the “flashbacks,” “dreams,” and “intrusions” variables.^5^

### 7.5 The bgms software

We have implemented the proposed Bayesian methods in the R package bgms, which can be installed from CRAN (see https://cran.r-project.org/web/packages/bgms/index.html). The Supplementary Material contains a small tutorial R script for using the package and redoing our reanalysis of the PTSD data. The computational aspects of the software are mostly written in C++ using the Rcpp package (Eddelbuettel, 2013). In addition to updating the computational algorithms described above and streamlining the analysis, we will investigate automated checks for convergence of the Markov chain. Ultimately, we plan to integrate the package into the JASP software (Love et al., 2019; Marsman & Wagenmakers, 2017; Wagenmakers, Love, et al., 2018a) so that applied researchers without R experience can also use the methodology.

## 8 Conclusion

We proposed an ordinal Markov random field model that takes into account the fact that most cross-sectional psychological data are measured on ordinal scales. We also introduced Bayesian methodology, including an implementation in the bgms package, to analyze the ordinal MRF with empirical data. This provides researchers with a clear way to assess the uncertainty of estimated network structures. We hope that by providing an appropriate network model for ordinal data and a natural uncertainty quantification for its estimates, we can help put the growing network literature on a more solid methodological footing.

## Figures and Tables

**Figure B1:**
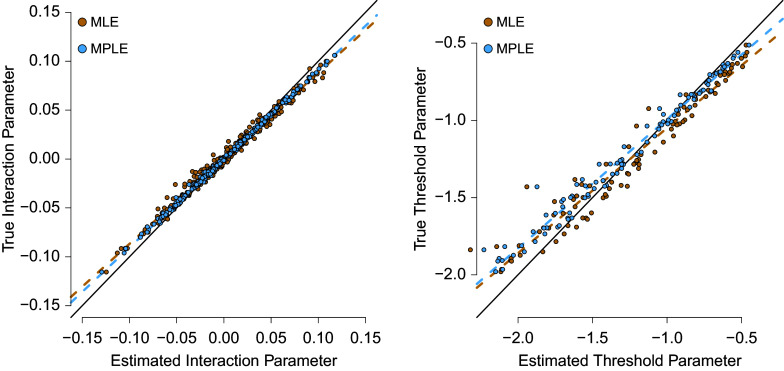
Scatterplots comparing MLEs (maximum likelihood estimates) and MPLEs (maximum pseudolikelihood estimates), each averaged across 500 datasets, against the values of the data-generating parameters. The left panel presents the estimates for pairwise interaction parameters, while the right panel focuses on the estimates for category threshold parameters. Each scatterplot features a solid line representing a perfect match (with an intercept of zero and a slope of one) and dashed lines depicting the actual regression lines.

**Figure E1:**
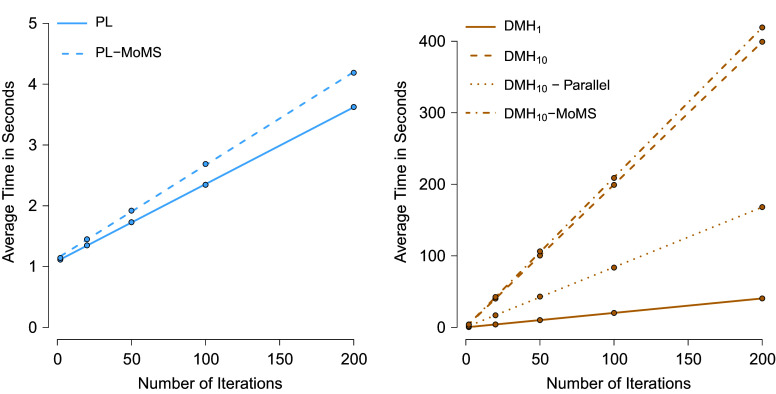
The left panel shows scatterplots illustrating the relationship between the average time (in seconds) to complete different implementations of the pseudolikelihood-based Gibbs sampler and the number of iterations. The right panel shows similar scatterplots for the DMH-based approach. In both panels, the straight lines represent regression lines modeling this relationship.

## Footnotes

1 Here, we also interpret extensions of the ordinal MRF that include higher order interactions as belonging to the same modeling family.

2 To simplify the notation, we assume a fixed number of response categories for the binary and ordinal variables. However, the model and accompanying software allow for a varying number of categories for the different variables in the network.

3 We take the variance of the normal distribution to be *n* times the diagonals of  , minus the inverse of the Hessian matrix for the edge weights of the pseudolikelihood.

4 In an earlier version of our article, we set the variance  equal to the asymptotic variance (i.e., the curvature at the posterior mode). This is currently the default in the bgms software, but this option cannot be used for and compared with DMH. However, starting with version 0.1.1 of the bgms software, there is an option to use an adaptive Metropolis–Hastings approach (Atchadé & Rosenthal, 2005; Griffin & Steel, 2022) and to tune the variance with a Robbins–Monro algorithm (Robbins & Monro, 1951). Specifically, in iteration *t* of the Gibbs sampler, we set the logarithm of the proposal variance  equal to

where  was the acceptance probability in the previous iteration and the target acceptance probability is  , which is optimal for a Metropolis-Hastings random walk in several scenarios. The parameter  , with  , scales the rate of adaptation. We set  to  .

5 The output of our Gibbs sampler lends itself naturally to the construction of such tests. In bgms, use save = TRUE to get the raw MCMC output.

## A The proposed distribution is a Markov random field

One way to show that the probability distribution in Eq. (2) is a Markov random field that satisfies so-called Markov properties. These properties derive conditional independence conditions from the structure of a graph (i.e., the presence or absence of an edge between two variables in the graph), captured here by the pairwise interaction matrix  . We first show that the probability distribution in Eq. (2) satisfies the global Markov property and then show that the local and pairwise Markov properties readily follow.

Since it is clear that the category thresholds do not affect the associations between variables, we set their values to zero here without loss of generality. Let  denote the set of response variables or vertices of the graph. We divide this set into three nonoverlapping sets:  . The matrix of pairwise associations is then characterized as

where  denotes the pairwise associations between variables  , and  denotes the pairwise associations between variables  and  . Let  be the vector of responses for the variables  . Then the probability distribution in Eq. (2) can be reformulated as

The global Markov property states that any two subsets of the variables  and  are conditionally independent, given a separating subset  :

thus, any path from a variable in  to a variable in  passes through one or more variables in  . This implies that there are no direct links or edges between  and  , and thus

All we need to show is that, in this case, the conditional distribution of the two separated sets factors as follows:

Assuming that  , the conditional distribution  is

Which is what we needed to show.

The global Markov property is stronger than the local and pairwise Markov properties, and they follow easily from the proof above. The pairwise Markov property states that any two variables *i* and *j* for which  are conditionally independent given the remaining variables:

Here,  ,  , and  . The local Markov property states that any variable *i* is conditionally independent of all other variables given its neighbors:

where  denotes the set of neighbors of variable *i*:

Note that this excludes *i* itself, since  . Here,  ,  , and  .

## B Assessing bias in maximum likelihood and pseudolikelihood estimates

The pseudolikelihood provides a practical approximation to the full likelihood, facilitating easier computations. We suspect that maximum pseudolikelihood estimates (MPLEs) are similar to maximum likelihood estimates (MLEs) in their ability to recover the underlying model parameters. This similarity was demonstrated by Keetelaar et al. (2024) in their study of the Ising model. Our goal is to investigate whether this property extends to the ordinal MRF. Keetelaar and colleagues also found that recovering pairwise interaction parameters is generally easier than recovering threshold parameters. As the number of category threshold parameters increases, which reduces the amount of information available for each, we expect that estimating these thresholds in the ordinal MRF could be more challenging.

In our evaluation of the Bayesian edge selection method in Section 5.2, we copy an analysis setup used by Park et al. (2022), which allows a direct comparison between our method and theirs as applied to the Ising model. To maintain consistency between our simulations and other numerical illustrations, we used 24 variables (  ) and 300 observations (  ) throughout. Our focus here is on ordinal variables with five response categories, hence  , while Park et al. (2022) and Keetelaar et al. (2024) focused on binary outcomes, i.e.,  . For each variable in our simulations, we generate four threshold parameters from a uniform distribution ranging from  to  , following the approach of Park et al., and sort the parameters in decreasing order. In this simulation, where edge selection is not an issue, a complete graph assumption is made. The pairwise interaction parameters are sampled from a normal distribution with a mean of zero and a standard deviation of  . Attempts to increase this standard deviation resulted in data sets with zero variance in certain variables, which was undesirable. Using these simulated parameter values, we generate 500 datasets from the ordinal MRF.

Due to the unavailability of MLEs in closed form and the computational intractability of the likelihood’s derivatives, traditional numerical methods such as Newton’s method or gradient descent are not feasible for our analysis. Therefore, we use the Robbins–Monro algorithm as an alternative approach (Robbins & Monro, 1951). This algorithm, when used to estimate a parameter  , iteratively updates the value of  as follows:

where  is a positive constant, and  is a stochastic approximation of the log likelihood’s derivative at  . In the context of the ordinal MRF, which belongs to the exponential family, the derivative of the log likelihood for any model parameter  can be expressed as

where  denotes the observed value of the sufficient statistic. For example, for the category threshold  it is  and for the pairwise interaction  it is  . The term  is the expected value of the sufficient statistic under the current model parameter estimates. Since this expected value does not have a closed-form solution and is difficult to compute numerically due to the presence of the intractable normalizing constant, we resort to (Markov chain) Monte Carlo methods for its estimation.

In our numerical experiments, we used the MPLEs as starting values for the Robbins–Monro algorithm for each of the 500 datasets. During these experiments, we tested different values for the algorithm’s tuning parameter,  . We found that a value of  worked well for the threshold parameters, while a much smaller value of  worked better for the pairwise interaction parameters. To compute the expected values needed in the algorithm, we used the final states of 200 separate Gibbs samplers, each run for 200 iterations. We ran the Robbins–Monro algorithm until the difference between successive estimates fell below  . To ensure computational feasibility, we also set an upper limit of 150 iterations for each run of the algorithm.

For each dataset, MPLEs were estimated using Newton’s method, while MLEs were estimated using the previously described Robbins–Monro algorithm. The data simulation for the Robbins–Monro algorithm was implemented in C++. For each of the datasets, we used Newton’s method to estimate the MPLEs. The MLEs, on the other hand, were computed using the Robbins–Monro algorithm as described above. The implementation of the data simulation for the Robbins–Monro algorithm was done in C++. In terms of computational efficiency, the process of calculating the MLEs was relatively fast, taking about 1 second on a single core of a MacBook Pro with an M1 chip. However, running the 150 iterations of the Robbins–Monro algorithm was more time consuming, taking about 45 min.

To evaluate the bias in the MPLEs and MLEs, we computed their average across all datasets. Figure B1 shows scatterplots that compare these averaged estimates to the parameter values used for generating data. The scatterplot in the left panel illustrates the pairwise interaction parameters, while the right panel focuses on the category threshold parameters. In these plots, dashed lines represent regression lines.

Note that the MPLEs for the pairwise interaction parameters are very similar to their MLE counterparts, as indicated by the nearly overlapping regression lines. However, there is a noticeable difference in variability, with MLEs showing greater variation compared to MPLEs. The comparison of category thresholds presents a different scenario. In contrast to the pairwise interaction parameters, the recovery of the category thresholds is less precise. Here, the scatterplot shows noticeable differences between MLEs and MPLEs. Interestingly, MPLEs tend to perform slightly better than MLEs in this respect, a detail that becomes clear when examining the regression lines.

It is also important to note that both MLEs and MPLEs exhibit bias in the estimation of both sets of parameters. However, the performance of MPLEs is similar to that of MLEs in terms of bias, suggesting that MPLEs are a viable and convenient alternative for estimating these model parameters.

## C The double metropolis–hastings algorithm

If we can generate observations directly from the probability model used for the likelihood based on a particular set of parameter values, we can bypass the computation of the model’s normalization constant using a clever implementation of the Metropolis–Hastings algorithm. Suppose

is a probability model for *x* with parameters  , a tractable kernel  , and  its normalizing constant. Suppose further that we are able to sample i.i.d. from the model, i.e.,  for some value  of the parameter. Let  be the prior distribution for the model parameter, and let  be a conditional proposal distribution based on the current state  of the Markov chain, e.g., a normal random walk Metropolis. Suppose we sample a proposed parameter value  and use it to generate an auxiliary data set  . The proposed value  in the pair  is a draw from the posterior distribution  . Murray et al. (2012) cleverly used this idea to design a Metropolis algorithm that has the desired posterior  as its invariant distribution. When we sample a proposed value from the posterior  , it is accepted with probability

which depends only on the tractable model kernels. This is called the *single variable exchange (SVE) algorithm*. Marsman, Maris, Bechger, and Glas (2017) and Marsman, Bechger, and Maris (2022a) showed how SVE can be adapted to efficiently sample from the posterior distributions of many random effects at once, such as the posterior distribution of ability in an IRT context.

The main drawback of SVE is that it requires us to be able to sample values *x* directly from the model  . Unfortunately, this is not possible for the ordinal MRF we are proposing. However, we can sample data from the model using a Gibbs sampler. Liang (2010) proposed the DMH algorithm in this case, which uses a Metropolis or Gibbs sampling algorithm to generate the auxiliary data. Liang used the detailed balance property to show that when we sample the auxiliary data in this way, the acceptance probability for DMH is the same as for SVE, regardless of the number of iterations *T* used by the Metropolis or Gibbs sampling approach to generate the auxiliary data. Of course, the quality of the approximation depends on *T*. As a rule of thumb, Liang suggests setting *T* equal to the dimension of  , i.e.  . As a naming convention, we call the Gibbs sampler used to generate auxiliary data the *inner Gibbs sampler*, and the Gibbs sampler used to sample from the posterior distribution of the model parameters the *outer Gibbs sampler*.

Although the DMH approach has been shown by Park and Haran (2018) to be the most efficient among MCMC methods for intractable distributions, it is extremely computationally intensive compared to the pseudolikelihood approach. The main computational bottleneck is the need to generate auxiliary data using the *inner Gibbs sampler* for each parameter of the model in each iteration of the *outer Gibbs sampler*. The nesting of the two Gibbs samplers explodes the computational time. We illustrate this problem in the runtime analysis in Appendix E.

## D The DMH-MoMS Gibbs sampler

Park et al. (2022) proposed a DMH approach to Bayesian edge selection for the Ising model using a continuous spike and slab prior. Although their proposed methodology is accompanied by some R and C++ software, it is unfortunately out of date and no longer functional. Because of this, and because, as we discussed in Section 3, Bayesian edge selection using a continuous spike and slab prior is not model selection consistent, we consider here a DMH-MoMS Gibbs approach to Bayesian edge selection using a discrete spike and slab prior. The DMH-MoMS Gibbs sampler is implemented in the R package dmhBGM (Marsman et al., 2024), which is available on Github: https://github.com/MaartenMarsman/dmhBGM.

The design of the proposed Gibbs sampler using DMH to bypass the computation of the intractable normalization constant is similar to that of the proposed Gibbs sampler using the pseudolikelihood. It includes two blocks of parameters to be updated

and we update the category thresholds one by one and the  pairs one by one. As with the Gibbs sampler for the pseudolikelihood approach, we follow these two block updates with an update of the interaction parameters for the currently active edges (i.e., we update  for which  ).

**Block I: Updating the category thresholds.** To update the category thresholds, we need to sample from a conditional distributions of the form

for  , and  . Here, we have used  ,

to denote the rest of the terms that are used in the exponent, and we have used  . Observe that the  and  are sufficient statistics.

To simulate from this conditional distribution, we use DMH and, as for the interaction parameters in the pseudolikelihood approach, propose a new parameter value from a normal density centered on the current state of the parameter, i.e., a random walk. As before, we use an adaptive Metropolis–Hastings approach (Atchadé & Rosenthal, 2005; Griffin & Steel, 2022) and tune the variance using a Robbins–Monro algorithm (Robbins & Monro, 1951).

When we sample  as a proposed value for  which currently has state  , say, and use that to simulate a new dataset  using the *inner Gibbs sampler* with *T* iterations, it is accepted with probability

where we have used  .

**Block II: Updating the edge indicators and interactions.** To update the edge indicator and the interaction parameter pair, we embed DMH in the MoMS procedure; we first propose a new state for the edge indicator  and use it to generate a proposed state for the association parameter  ; if  , we propose  , and if  , we sample  from a normal density centered on the current state  . The DMH approach adds an auxiliary variable step, and we sample the auxiliary data  based on the proposed state  .

Let  and  denote the current state of the edge indicator and the interaction between a variable *i* and *j*, let  and  denote its proposed state, and let  denote the auxiliary data. We accept the proposed states with probability

where  is the current state of the pairwise interaction matrix, and  is the proposed state, with elements in row *i*, column *j*, and row *j*, column *i*, set equal to the proposed value  . If  , we propose  and  , and accept the proposed values with probability

Conversely, if  , we propose  and  . We accept  and  with probability

As in the pseudolikelihood MoMS Gibbs sampler, after updating the edge indicator and interaction parameter pair, we perform an additional Metropolis–Hastings update step for the interaction parameters for the edges currently in the model using DMH (i.e., we update  for which  ). This additional step, which helps improve the convergence rate of the MoMS algorithm (see the main text for details), also allows us to perform the Robbins–Monro procedure to adjust the variance of the proposed distribution for the interaction effects.

## E Runtime analysis

Following our analysis of the operational characteristics of the pseudolikelihood and DMH-based approaches, we now turn to a comparative assessment of their running times. In order to establish a robust measure of running time, we conducted a series of experiments using the Gibbs samplers, both with and without the selection process. Specifically, each variant of the Gibbs sampler was run ten times over a range of iterations—2, 20, 50, 100, and 200—using one of the datasets previously used in our bias assessment of the parameter estimates. For these runtime experiments, the microbenchmark R package (Mersman, 2023) was used to ensure a consistent and controlled computational environment. All procedures were run on a single-core MacBook Pro equipped with an M1 Pro chip to provide a standard baseline for comparing the performance of the two approaches.

The results of our runtime experiments are shown in Figure E1. One observation from these results is the almost perfect linear relationship between the runtimes and the number of iterations performed by the Gibbs sampler. To quantify this relationship, we performed a regression of the runtime (in seconds) against the number of iterations, with the detailed regression results presented in Table E1. Based on these regression estimates, we can infer that for 100,000 iterations, the pseudolikelihood approach would take about 21 min to estimate the posterior distribution without selection, while the DMH approach with ten iterations of the inner Gibbs sampler would take about 55 hr, a bit over two days. In the case of the two MoMS Gibbs samplers, the pseudolikelihood approach is estimated to take about 26 min and the DMH algorithm with ten iterations of the inner Gibbs sampler about 58 hr, about two and a half days.

**Table E1:** This table presents the regression coefficients obtained from analyzing the total runtime (in seconds) against the number of iterations for various implementations of the Gibbs sampler

Gibbs sampler	Intercept	Slope	Expected runtime (hours) 100K iterations
PL	1.092	0.013	0.36
PL-MoMS	1.137	0.015	0.42
DMH	0.028	0.202	5.61
DMH	0.289	1.994	55.39
DMH (6 cores)	0.249	0.839	23.31
DMH -MoMS	0.386	2.094	58.17

*Note*: The rightmost column specifically quantifies the expected runtime in hours for each method when run for  iterations, providing a direct comparison of their computational demand.

An interesting point to note is the similarity in runtimes between these Gibbs samplers and those used for estimation, even though the latter require sampling from an additional full conditional distribution. But in the MoMS Gibbs samplers, we only need to sample from this additional full conditional distribution, the posterior distribution of the pairwise interaction parameter, when the corresponding edge is present (i.e.,  ). Thus, the similarity in runtime may be due to the fact that most effects are estimated to be absent, which means that we only need to sample from the full conditional posterior of a few pairwise interaction parameters.

The duration of each iteration in the inner Gibbs sampler appears to have a direct linear effect on the overall runtime. For example, our analysis predicts that 100,000 iterations of a Gibbs sampler using the DMH algorithm with only a single iteration in its inner Gibbs sampler would take approximately 5.6 hr to complete. This finding implies a proportional relationship between the number of iterations in the inner Gibbs sampler and the total runtime: reducing the number of iterations in the inner Gibbs sampler by a factor of ten results in a tenfold reduction in runtime. Extrapolating from this, we can conclude that 100,000 iterations of a Gibbs sampler using the DMH algorithm with  iterations in its inner Gibbs sampler would take a considerable amount of time, about 4.5 years. This projection underscores the significant time required by the DMH algorithm, especially when many iterations are used for its inner Gibbs sampler.

Parallelizing certain components of the DMH algorithm can play a crucial role in reducing its computational load. To investigate this, we used the RcppParallel package (Allaire et al., 2023) for parallel processing in the auxiliary data generation phase of DMH. When we run 100,000 iterations of the Gibbs sampler using the DMH algorithm with ten iterations in its inner Gibbs sampler on six cores, the total runtime is significantly reduced to about 23 hr. Although this is not a sixfold speedup, but just over a twofold speedup, it still represents a 60% reduction in runtime compared to performing the same computation on a single core. This significant speedup illustrates the effectiveness of parallelization in improving the computational efficiency of the DMH algorithm.

## F Prior robustness analysis

In Figure F1, we plot the estimated inclusion Bayes factors under the unit information prior against the Cauchy  prior (left panel) and the Cauchy  prior (right panel). We focused the plots on the interval between  and  , which contains the most results. The black diagonal line in each panel passes through the origin and has a unit slope. The blue diagonal line in the right panel does not go through the origin, but through minus the logarithm of the Cauchy scale (i.e.,  ). Note that the log Bayes factors are very similar under the Cauchy  prior and the unit information prior. The log Bayes factors have a slightly higher value of  on average under the unit information prior (i.e., the inclusion Bayes factors are about  times larger under the unit information prior). Furthermore, the log Bayes factors appear to be sensitive to the scale value of the Cauchy density; shrinking the scale moves the evidence toward edge inclusion, while increasing the scale moves the evidence toward edge exclusion. That increasingly diffuse priors lead to an increase in evidence for the null hypothesis is well known (Jeffreys, 1961; Lindley, 1957) and results from the increasingly extreme values supported by the prior but not by the data. The log Bayes factors under the Cauchy  prior are on average  smaller than under the unit information prior (i.e., the inclusion Bayes factors are about  times larger under the unit information prior). Additional analyses revealed that the relationship between the Bayes factors under the unit information prior and the Cauchy prior becomes more diffuse as scale increases.
